# Regulatory functions of AcuK and AcuM transcription factors in fungal metabolic adaptation, stress response, and virulence

**DOI:** 10.3389/fcimb.2025.1717070

**Published:** 2025-12-08

**Authors:** Tanaporn Wangsanut, Nopawit Khamto, Hamid Alinejad-Rokny, Monsicha Pongpom

**Affiliations:** 1 Department of Microbiology, Faculty of Medicine, Chiang Mai University, Chiang Mai, Thailand; 2 Department of Biochemistry, Faculty of Medical Science, Naresuan University, Phitsanulok, Thailand; 3 UNSW BioMedical Machine Learning Lab (BML), School of Biomedical Engineering, UNSW, Sydney, NSW, Australia

**Keywords:** AcuK, AcuM, fungi, virulence, iron, hypoxia, stress response, adaptation

## Abstract

Fungal species thrive in diverse ecological niches and must dynamically adjust their metabolism, growth, and development in response to environmental fluctuations. Transcriptional regulation plays a pivotal role in these adaptive processes, with Zn_2_Cys_6_ transcription factors constituting the largest family of fungal-specific regulators that orchestrate metabolism, development, and pathogenicity. Among these, AcuK and AcuM represent a distinct subfamily with diverse key regulatory functions. In this review, we provide a comprehensive analysis of AcuK and AcuM functions across various fungal species, including major human pathogens (*Aspergillus fumigatus, Candida albicans, Cryptococcus neoformans, Talaromyces marneffei*) and model saprophytic fungi (*Aspergillus nidulans, Neurospora crassa, Podospora anserina, Saccharomyces cerevisiae*). Our analysis reveals that AcuK and AcuM regulate core metabolic pathways, particularly alternative carbon utilization via gluconeogenesis (e.g., *acuG* encoding fructose-1,6-bisphosphatase and *acuF* encoding phosphoenolpyruvate carboxykinase) and alternative respiration (e.g., *aox* encoding alternative oxidase). Beyond metabolic regulation, AcuK and AcuM play crucial roles in a host-induced stress adaptation, especially responses to iron limitation, hypoxia, and host immune interactions—factors that are critical for fungal pathogenicity. Additionally, these transcription factors influence nitric oxide detoxification by modulating *YHB1*, a heme- and oxygen-dependent nitric oxide dioxygenase. Structural modelling of AcuK/AcuM heterodimer formation and DNA-binding interactions provides mechanistic insights into their regulatory functions. Understanding these transcriptional networks not only advances our knowledge of fungal adaptation but also highlights AcuK and AcuM as potential targets for antifungal therapy.

## Introduction

Fungi inhabit diverse ecological niches and must continuously adapt to fluctuating environmental conditions. During infection, pathogenic fungi encounter a broader range of host-derived stresses, including temperature changes, nutrient scarcity, hypoxic conditions, and host immune defenses. For example, the host environment imposes iron limitation as a defense strategy to restrict fungal growth and virulence (Hernández-Chávez et al., 2017; Jia et al., 2024). Additionally, specific infection sites may present glucose-deprived and hypoxic conditions, further challenging fungal metabolism (Puerner et al., 2023; Tucey et al., 2018). To counteract hostile conditions, fungi have evolved sophisticated transcriptional regulatory mechanisms to coordinate stress tolerance, metabolic adaptation, and cellular homeostasis.

AcuK and AcuM are members of the Zn_2_Cys_6_ transcription factor family. They are characterized by a well-conserved CysX_2_CysX_6_CysX_5-12_CysX_2_CysX_6-8_Cys motif within their N-terminal DNA-binding domain (MacPherson et al., 2006). This zinc-coordinated structure ensures proper protein folding and facilitates DNA recognition (Vallee et al., 1991). The *acuK* and *acuM* genes were initially identified in a genetic screening of non-pathogenic fungus *Aspergillus nidulans* mutants that were unable to metabolize acetate as an alternative carbon source, leading to their classification as acetate utilization (*acu*) genes (Armitt et al., 1976). Mutants lacking *acuK* or *acuM* exhibited severe growth defects on all gluconeogenic carbon sources, confirming that these genes are essential for gluconeogenesis. Specifically, the expression of key gluconeogenic enzymes—including fructose-1,6-bisphosphatase (*acuG*), phosphoenolpyruvate carboxykinase (*acuF*), and enolase (*acuN*)—was significantly reduced in *acuK* and *acuM* mutants (Armitt et al., 1976). Mechanistically, AcuK and AcuM function as transcriptional activators by forming homo- or heterodimeric complexes at target gene promoters (Suzuki et al., 2012; Hynes et al., 2007). Thus, AcuK and AcuM serve as key regulators of gluconeogenesis in *A. nidulans*. Interestingly, subsequent studies in human fungal pathogens *Aspergillus fumigatus* have unexpectedly demonstrated their involvement in iron acquisition and pathogenesis (Liu et al., 2010; Pongpom et al., 2015).

As AcuK and AcuM proteins regulate multiple distinct pathways in *Aspergillus* species, this prompts us to analyze the functions of AcuK and AcuM homologs across diverse fungal species. This review presents the first comprehensive analysis of the current knowledge on AcuK and AcuM functions by collecting genes encoding homologs of these transcription factors whose functions have been experimentally verified. Phylogenetic analysis suggested that the metabolic functions of these transcription factors have likely evolved differently between pathogenic and non-pathogenic fungi. We highlight the common role of AcuK and AcuM in controlling gluconeogenesis and various host stress responses in both non-pathogenic and pathogenic fungi. The distinct roles of AcuK and AcuM homodimers, including iron assimilation and hypoxia responses, were summarized and proposed to be related to pathogenic traits in pathogenic fungi. Although the mechanism of cross-regulation of metabolic pathways remains unknown, these transcription factors could be potential therapeutic targets due to their versatile roles in regulating metabolism and virulence traits.

## Identification of AcuK and AcuM homologs in fungi

**Figure 1:**
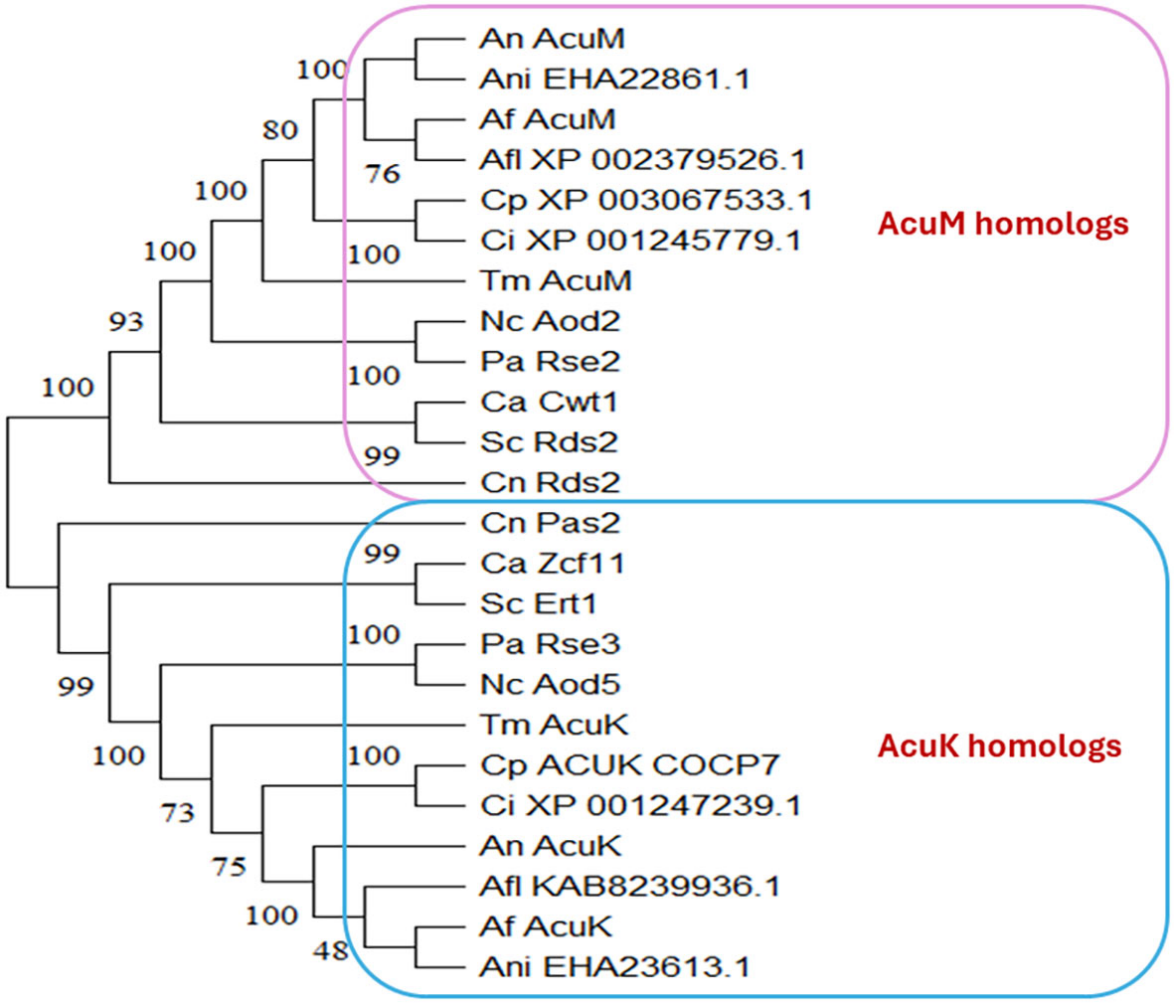
Phylogenetic tree of AcuK and AcuM homologs in fungal species. A maximum likelihood phylogenetic tree of the AcuK and AcuM proteins was constructed using MEGA 11 software with a bootstrap value of 1000 [9]. Af, *Aspergillus fumigatus*; An, *Aspergillus nidulans*; Ani, *Aspergillus niger*; Afl, *Aspergillus flavus*; Ca, *Candida albicans*; Cn, *Cryptococcus neoformans;* Ci, *Coccidioides immitis*; Cp, *Coccidioides posadasii*; Nc, *Neurospora crassa*; Pa, *Podospora anserina*; Sc, *Saccharomyces cerevisiae*; Tm, *Talaromyces marneffei*.

**Figure 2:**
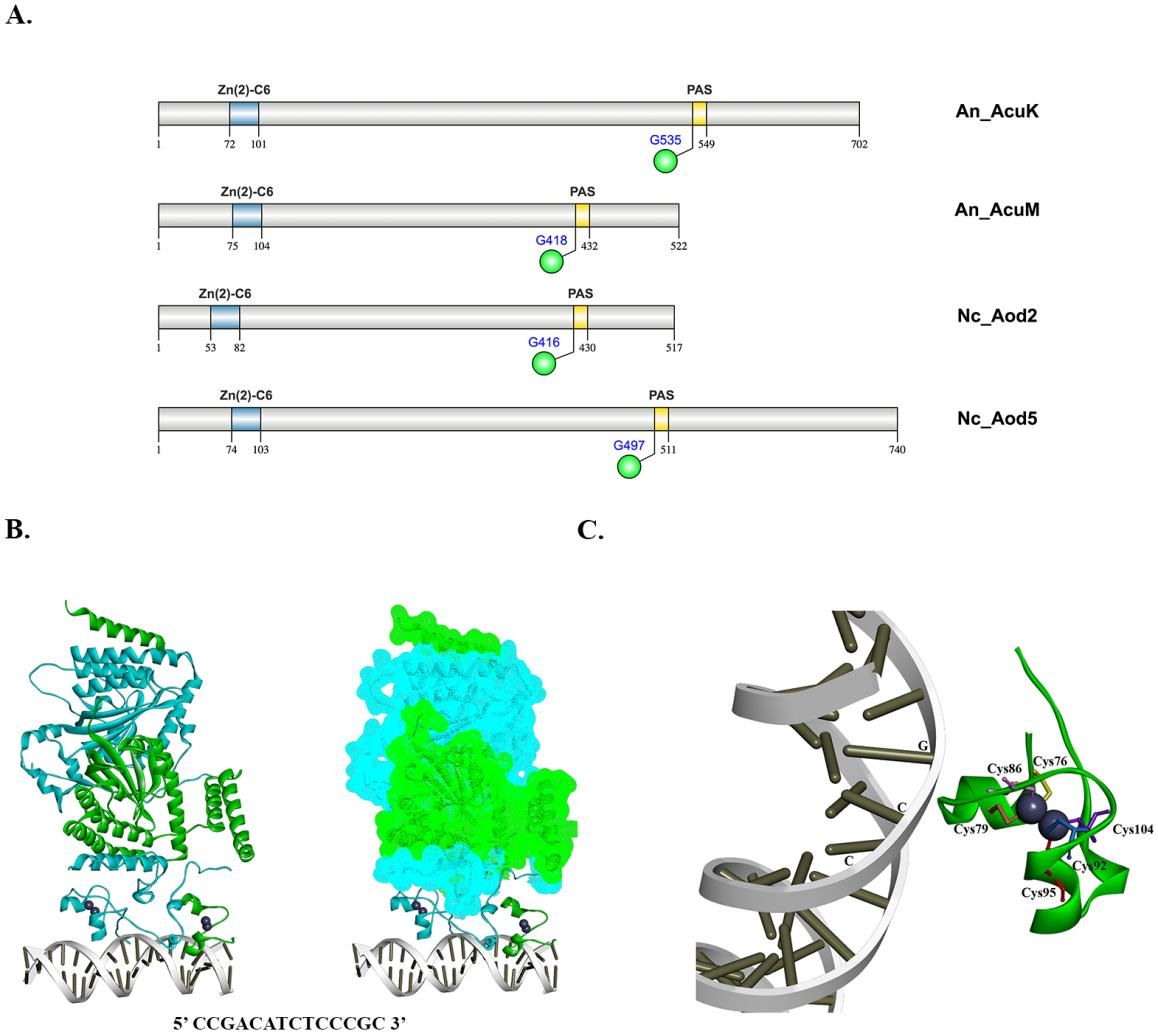
Key regulatory elements of AcuK and AcuM transcription factors. **(A)** Functional domains of AcuK and AcuM homologs from representative fungal models are depicted (https://ibs.renlab.org/) (Xie et al., 2022). Abbreviation: Zn(2)-C6: Zn_2_Cys_6_ DNA binding domain; PAS: PAS domain, green sphere indicates residues that have been experimentally validated to be important for their functions. **(B)** Model of AcuK, AcuM, and *acuF* promoter (5’ CCGACATCTCCCGC 3’) complex formation from *A*. *nidulans* is illustrated (https://alphafoldserver.com/) (Abramson et al., 2024). Blue, AcuK; Green, AcuM; Grey, DNA; Purple sphere, Zn^2+^. **(C)** Zn_2_Cys_6_ DNA binding domain of AcuM from *A*. *nidulans* is highlighted.

Historically, different studies have assigned distinct names to these homologous proteins, despite their conserved protein sequences and functional domain similarities. To systematically identify AcuK and AcuM homologs across fungal taxa, we conducted a protein BLAST homology search and phylogenetic tree analysis (**Figure 1**). Most of the analyzed fungal species contain both AcuK and AcuM homologs, which cluster separately in the phylogenetic tree, indicating a conserved evolutionary divergence between these two transcription factors. Structurally, all AcuK and AcuM proteins share a well-conserved zinc cluster domain, characteristic of Zn_2_Cys_6_ transcription factors. Additionally, they possess a Per-Arnt-Sim (PAS) domain, defined by the G-X_1_-I-X_3_-N-X_7_-G motif, which is implicated in environmental sensing and protein-protein interactions (**Figure 2**).

## The AcuK and AcuM regulatory functions

Regulatory functions of AcuK and AcuM homologs that have been experimentally validated to date are summarized in **Table 1**. Common AcuK and AcuM target genes identified by Chromatin immunoprecipitation (ChIP) assays or gene expression analyses are shown in **Tables 2**, **3**, respectively.

**Table 1:** Summary of AcuK and AcuM function across fungal species.

Species	AcuK homologs		AcuM homologs	
	Protein name (Ref)	Function*	Protein name (Ref)	Function
Non-pathogenic fungi				
*Saccharomyces cerevisiae*	Ert1 (Gasmi et al., 2014)	GCN	Rds2 (Soontorngun et al., 2007)	GCN, OS, OX
*Aspergillus nidulans*	AcuK (Hynes et al., 2007)	GCN, AOX	AcuM (Hynes et al., 2007)	GCN, AOX
*Neurospora crassa*	Aod5 (Chae et al., 2007)	GCN, AOX, FHB	Aod2 (Chae et al., 2007)	GCN, AOX, FHB
*Podospora anserina*	Rse3 (Sellem et al., 2009)	GCN, AOX, FHB	Rse2 (Sellem et al., 2009)	GCN, AOX, FHB
Pathogenic fungi				
*Candida albicans*	Zcfl1 (Liu et al., 2023)	GCN, AOX	Cwt1 (Moreno et al., 2003)	GCN, AOX, FHB, OS, OX, HPI
*Cryptococcus neoformans*	Pas2 (Zhao and Lin, 2021)	GCN, AOX, FHB, OS, Fe, HYP	Rds2 (Zhao and Lin, 2021)	HYP
*Talaromyces marneffei*	AcuK (Amsri et al., 2021)	GCN, AOX, FHB, Fe, HPI	AcuM (Wangsanut et al., 2024)	GCN, AOX, FHB, OX, Fe, HPI
*Aspergillus fumigatus*	AcuK (Pongpom et al., 2015)	GCN, AOX, Fe, HPI	AcuM (Liu et al., 2010)	GCN, AOX, FHB, Fe, HPI

GCN, Gluconeogenesis; AOX, Alternative oxidase; FHB, Flavohemoglobin/nitric dioxygenase/Nitrosative stress; OS, Other stress (Heat, osmotic, drug, cell wall stressors); OX, Oxidative stress; Fe, Iron-dependent metabolism; HYP, Hypoxic response; HPI, Host-pathogen interaction/Virulence in animal models/Macrophage interaction.

**Table 2:** Common AcuK and AcuM target genes in fungal species.

Target	Organism	Lifestyle	Gene ID	Gene expression in mutants/Binding pattern	Ref
Fructose-1, 6-biphosphate (*fbp*)	*T. marneffei*	Pathogen	PMAA_041280	Down (qRT-PCR)	Wangsanut et al., 2024
	*A. fumigatus*	Pathogen	Afu4g11310	Down	Liu et al., 2010
	*N. crassa*	Mold	NCU04797	Down	Qi et al., 2017
	*P. anserina*	Mold	Pa_4_9360	Down	Bovier et al., 2014
	*A. nidulans*	Mold	AN5604.3	Down	Suzuki et al., 2012
	*C. albicans*	Pathogen	C3_07830W	Binding (ChIP)	Sellam et al., 2012
Phosphoenolpyruvate carboxykinase (*pepck*)	*T. marneffei*	Pathogen	PMAA_032350	Down (RNA-seq)	Wangsanut et al., 2024
	*A. fumigatus*	Pathogen	Afu6g07720	Down	Liu et al., 2010
	*N. crassa*	Mold	NCU09873	Down	Qi et al., 2017
	*P. anserina*	Mold	Pa_4_3160	Down	Bovier et al., 2014
	*A. nidulans*	Mold	AN1918.3	Down	Suzuki et al., 2012
	*C. albicans*	Pathogen	CR_00200W	Down/Binding (ChIP)	Sellam et al., 2012
	*C. neoformans*	Pathogen	CNAG_04217	Down	Zhao and Lin, 2021
High-affinity iron permease (*ftrA*)	*T. marneffei*	Pathogen	PMAA_057440	Up	Wangsanut et al., 2024
	*A. fumigatus*	Pathogen	Afu5g03800	- Down	Liu et al., 2010
				- Unaffected	Caballero et al., 2024
	*C. albicans*	Pathogen	C1_14130W	Down/Binding (ChIP)	Sellam et al., 2012
	*C. neoformans*	Pathogen	CNAG_06242	Down	Zhao and Lin, 2021
Multicopper ferroxidase (*fetC*)	*T. marneffei*	Pathogen	PMAA_057450	Up	Wangsanut et al., 2024
	*A. fumigatus*	Pathogen	Afu5g03790	Na	
	*C. albicans*	Pathogen	C6_00440C	Down/Binding (ChIP)	Sellam et al., 2012
Alternative oxidase (*aox*)	*T. marneffei*	Pathogen	PMAA_029240	Down (RNA-seq)	Wangsanut et al., 2024
	*A. fumigatus*	Pathogen	Afu2g05060	Down	Liu et al., 2010
	*N. crassa*	Mold	NCU07953	Down	Qi et al., 2017
	*P. anserina*	Mold	Pa_3_1710	Down	Bovier et al., 2014
	*A. nidulans*	Mold	AN2099.3	Down	Suzuki et al., 2012
	*C. albicans*	Pathogen	AF116872.1	Down	Liu et al., 2023
	*C. neoformans*	Pathogen	CNAG_00162	Down	Zhao and Lin, 2021
Alternative NADH dehydrogenase (pyridine nucleotide-disulfide oxidoreductase)	*T. marneffei*	Pathogen	PMAA_065210	Down (RNA-seq)	Wangsanut et al., 2024
	*A. fumigatus*	Pathogen	Afu1g17180	Down	Liu et al., 2010
	*N. crassa*	Mold	na	na	
	*P. anserina*	Mold	Pa_7_1820	Down	Bovier et al., 2014
	*A. nidulans*	Mold	na	Na	
	*C. albicans*	Pathogen	na	Na	
Malic enzyme	*T. marneffei*	Pathogen	PMAA_070640	Down (RNA-seq)	Wangsanut et al., 2024
	*A. fumigatus*	Pathogen	Afu2g08280	Down	Liu et al., 2010
	*N. crassa*	Mold	NCU03651	Down	Qi et al., 2017
	*P. anserina*	Mold	na	Na	
	*A. nidulans*	Mold	AN6168.3	*	Suzuki et al., 2012
	*C. albicans*	Pathogen	na	Na	
Cytochrome C	*T. marneffei*	Pathogen	PMAA_076160	Down (RNA-seq)	Wangsanut et al., 2024
	*A. fumigatus*	Pathogen	Afu2g13110	Down	Liu et al., 2010
	*N. crassa*	Mold	NCU01808	Down	Qi et al., 2017
	*P. anserina*	Mold	na	Na	
	*A. nidulans*	Mold	na	Na	
	*C. albicans*	Pathogen	na	Na	
Flavohemoglobin/Nitric oxide dioxygenase (*yhb*)	*T. marneffei*	Pathogen	PMAA_088690	Up (RNA-seq)	Wangsanut et al., 2024
	*A. fumigatus*	Pathogen	Afu8g06080	Up (Microarray)	Liu et al., 2010
	*N. crassa*	Mold	NCU10051	Up	Qi et al., 2017
	*P. anserina*	Mold	Pa_5_1700	Down	Bovier et al., 2014
	*A. nidulans*	Mold	na	Na	
	*C. albicans*	Pathogen	CR_O7790C	Up	Sellam et al., 2012
	*C. neoformans*	Pathogen	CNAG_01464	Down	Zhao and Lin, 2021

*presence of DNA-binding motif; na, not reported.

**Table 3:** Experimentally verified DNA binding motifs of the AcuK and AcuM homologs.

Organism	Homolog name	Gene	DNA binding motif sequences	Reference
*Aspergillus nidulans*	AcuK	*acuF* (phosphoenolpyruvate carboxykinase)	GCCCCAGCCGACATCTCCCG	Suzuki et al., 2012; Hynes et al., 2007)
	AcuM		GGTTCGGGTCTATTCGGGCTA	
*Candida albicans*	Cwt1	*YHB1* (Nitric oxide dioxygenase)	CAAGTTTAAAGACCGAGAGAATGA	Sellam et al., 2012)
	Cwt1 Zcf11	*AOX2* (Alternative oxidase)	CGATAATTTACCGGTTTGATTAATTAATTATACATCCCGTCATATTCCA	Liu et al., 2023)
*Saccharomyces cerevisiae*	Gsm1 Ert1 Rds2	*FBP1* (Fructose 1,6-bisphosphatase)	CCGGAGTTA	Martinez et al., 2024)
*Neurospora crassa*	Aod2 Aod5	*aod-1* (Alternative oxidase)	GGCACGGACAAACTCGGTGTT	Chae et al., 2007)

### Control of gluconeogenesis

The role of AcuK and AcuM in gluconeogenesis is evolutionarily conserved across the analyzed fungal species. Homologs of these proteins have been identified in filamentous ascomycetes, non-pathogenic yeasts, and pathogenic yeasts, where they regulate the expression of gluconeogenic genes. In filamentous fungi such as *A. nidulans, A. fumigatus*, and *Talaromyces marneffei*, deletion of *acuK* or *acuM* orthologous genes results in growth defects on non-glucose carbon sources, underscoring their conserved function in alternative carbon metabolism (Armitt et al., 1976; Suzuki et al., 2012; Hynes et al., 2007; Liu et al., 2010; Pongpom et al., 2015; Wangsanut et al., 2024; Amsri et al., 2021). Furthermore, expression of two key genes in gluconeogenesis, *fbp* and *pepck*, is dependent on AcuK and AcuM orthologs in *A. fumigatus, T. marneffei*, and *Podospora anserina* (Liu et al., 2010; Wangsanut et al., 2024; Bovier et al., 2014) (**Table 2**). AcuK and AcuM were shown by a ChIP assay to function as a heterodimer in *A. nidulans* (Suzuki et al., 2012).

In budding yeast (*Saccharomyces cerevisiae*), AcuK and AcuM homologs are Ethanol-Regulated Transcription Factor 1 (Ert1) (Gasmi et al., 2014) and Regulator of Drug Sensitivity (Rds2), respectively (Soontorngun et al., 2007). Additionally, a third Zn_2_Cys_6_ transcription factor, Glucose Starvation Modulator 1 (Gsm1), functions in conjunction with Ert1 and Rds2 (Van Bakel et al., 2008). These three paralogs work cooperatively to regulate genes involved in gluconeogenesis and metabolic reprogramming. Their heterodimeric interactions create a complex regulatory network for DNA binding and transcriptional activation (Martinez et al., 2024). For example, Gsm1 and Rds2 bind cooperatively at the *FBP1* promoter, while Ert1 and Rds2 regulate *HAP4*, which encodes a master regulator of mitochondrial respiration. Due to genetic redundancy, deletion of *ERT1, RDS2*, or *GSM1*, either individually or in combination, does not impair growth on non-fermentable carbon sources (Martinez et al., 2024).

In the pathogenic yeast *Candida albicans*, Zinc Cluster Transcription Factor 11 (Zcf11) and Cell Wall Transcription Factor 1 (Cwt1) are homologs of AcuK and AcuM (Liu et al., 2010, 2023; Moreno et al., 2007). Functional studies demonstrate that the *cwt1* mutant, but not the *zcf11* mutant, is defective in utilizing alternative carbon sources, such as lactate, citrate, acetate, and casamino acids, indicating that Cwt1 can function independently of Zcf11 in regulating carbon metabolism. Chromatin immunoprecipitation (ChIP-chip and ChIP-qPCR) analyses further confirmed that Cwt1 directly binds the promoters of key gluconeogenic genes (*PCK1, FBP1*, and *PGK1*) (Sellam et al., 2012). In *Cryptococcus neoformans*, AcuK and AcuM homologs are Pas2 and Rds2, respectively. Similarly, the expression of *pepck* (*CNAG_04217*) is dependent on Pas2, whereas the role of Rds2 in gluconeogenesis has yet to be characterized (Zhao and Lin, 2021). These findings underscore the conserved regulatory function of AcuK and AcuM homologs in fungal gluconeogenesis across fungal taxa.

### Control of alternative respiratory pathway

Alternative oxidase (AOX) serves as the terminal oxidase and plays important roles in maintaining mitochondrial metabolic and signaling homeostasis. AOX endows the respiratory system with flexibility in the coupling among the carbon metabolism pathway, electron transport chain (ETC) activity, and ATP turnover. AOX allows electrons to bypass the main cytochrome pathway, thereby limiting the generation of reactive oxygen species (ROS). The role of AOX in mitigating or averting oxidative stress is therefore important for fungal virulence (Tian et al., 2020).

In the filamentous fungal model, *Neurospora crassa*, Aod5 and Aod2 are the orthologs of AcuK and AcuM, respectively. These transcription factors have been independently identified as key regulators of *aod-1*, which encodes the AOX enzyme (Chae et al., 2007). Aod2 and Aod5 function as a heterodimer that binds the alternative oxidase induction motif (AIM) within the *aod-1* promoter to activate *aod-1* transcription (Chae et al., 2007). In fact, the *aox* gene is typically induced under conditions that inhibit the classical electron transport chain (ETC), such as exposure to chemical respiratory inhibitors like antimycin A or cyanide, or nitric oxide (NO) produced by host immune defense during microbial infection (Millenaar and Lambers, 2003). Accordingly, the *N. crassa aod-2* and *aod-5* mutants are unable to induce *aod-1* expression and fail to grow in the presence of antimycin A (Descheneau et al., 2005). In *P. anserina*, the Rse3 and Rse2 proteins—orthologs of AcuK and AcuM—were independently identified in a genetic screen for suppressors of long-lived respiratory mutants (*cox5::ble*, a Complex IV mutant, and *cyc1-1*, a Complex III mutant) (Sellem et al., 2009). Both *rse3* and *rse2* are required for *aox* gene expression, reinforcing their regulatory role in the alternative respiratory pathway (Sellem et al., 2009). Similarly, in *A. nidulans*, a*cuM* mutants exhibit increased sensitivity to antimycin A, and both transcription factors are essential for antimycin A-induced *aox* gene expression (Suzuki et al., 2012). These findings collectively demonstrate that AcuK and AcuM proteins are critical for AOX regulation in non-pathogenic filamentous fungi.

In *C. albicans*, recent studies revealed that *cwt1* and *zcf11* mutants are hypersensitive to antimycin A, and their *aox* gene expression is significantly downregulated in response to antimycin A exposure (Liu et al., 2023). The evidence suggests that Cwt1 and Zcf11 function as a heterodimer in AOX regulation, as double deletion of *CWT1* and *ZCF11* results in phenotypes identical to those of individual deletion mutants (Liu et al., 2023). In *A. fumigatus, C. neoformans*, and *T. marneffei*, the direct phenotypic effects of *acuK* and *acuM* mutations on antimycin A sensitivity have not been explicitly tested. However, transcriptomic analyses indicate that AOX gene expression is differentially regulated in these mutants, suggesting a conserved, distinct regulatory role of the homodimers (Liu et al., 2010; Wangsanut et al., 2024; Zhao and Lin, 2021) (**Table 2**).

Overall, the AcuK/AcuM family, potentially as heterodimers, plays a highly conserved role in the transcriptional regulation of *aox* genes across diverse fungal species. This regulatory function is independent of the fungi’s pathogenic lifestyle.

### Control of oxidative stress response

In the dimorphic fungus *T. marneffei*, AcuK and AcuM have been shown to play a role in oxidative stress resistance. The Δ*acuM* mutant is hypersensitive to both menadione and hydrogen peroxide, while the Δ*acuK* mutant shows sensitivity to hydrogen peroxide only (Wangsanut et al., 2024). Notably, the yeast form of both mutants exhibits more severe antioxidant defects compared to the mold form (Wangsanut et al., 2024). These findings suggest that AcuK and AcuM contribute to oxidative stress tolerance, particularly in the pathogenic yeast phase of *T. marneffei*. In contrast, the Δ*acuM* mutant from *A. fumigatus* is not sensitive to the oxidative stressor hydrogen peroxide, indicating that AcuM is dispensable for oxidative stress response in this fungal species (Liu et al., 2010).

The mechanisms by which the transcription factors AcuK and AcuM regulate responses to menadione and hydrogen peroxide have not been fully investigated in *T. marneffei*. Further studies are required to elucidate the underlying mechanisms in *T. marneffei* and to explore the conserved roles of AcuK and AcuM in regulating oxidative stress in other fungal species.

### Control of nitrosative stress response

Evidence showed that AcuK and AcuM homologs appear to regulate fungal responses to nitrosative stress in some fungal species.

Cwt1 has been identified as a major regulator of nitrosative stress resistance in *C. albicans* (Sellam et al., 2012). ChIP assays confirmed that Cwt1 directly binds the *YHB1* promoter, which encodes nitric oxide dioxygenase, a key enzyme involved in nitric oxide detoxification. Interestingly, *YHB1* expression is strongly upregulated in the Δ*cwt1* mutant, indicating that Cwt1 functions as a negative regulator of nitric oxide dioxygenase under nitrosative stress (Sellam et al., 2012). Strikingly, transcriptomic analyses suggest that AcuK and AcuM regulate *YHB1* homolog expression in multiple filamentous fungi (**Table 2**). Future experimental studies testing the role of AcuK and AcuM homologs in nitrosative stress resistance in other fungi could directly provide valuable insight into the regulatory functions of these regulators.

### Control of drug and cell wall stress response

In *S. cerevisiae*, the Δ*rds2* mutant exhibits hypersensitivity to the antifungal drug ketoconazole, indicating a role for Rds2 in drug resistance (Akache and Turcotte, 2002). In *C. albicans*, the transcription factor Cwt1 plays a crucial role in cellular adaptation to various environmental stressors. For instance, Cwt1 is required for cell wall integrity because the *Δcwt1* mutant displays increased sensitivity to Congo red and calcofluor white, while exhibiting enhanced resistance to SDS and zymolyase (Moreno et al., 2007, 2003). Analysis of the mutant’s cell wall composition revealed a decrease in β-glucan and a compensatory increase in mannan, correlating with differential expression of genes involved in cell wall biogenesis and structure (Moreno et al., 2007).

In contrast, the Δ*acuM* mutant from *A. fumigatus* exhibited no significant differences in environmental and oxidative stress responses compared to the wild-type strain when tested for susceptibility to cell wall/membrane stressors (Congo red, calcofluor white, SDS), the oxidative stressor hydrogen peroxide, and antifungal agents caspofungin and amphotericin B (Liu et al., 2010). In *C. neoformans*, the Δ*pas2* mutant displayed wild-type levels of resistance to high temperature (37 °C), osmotic stress (1.5 M NaCl), Congo red-induced cell wall stress, and UV radiation, but showed mild sensitivity to the broad-spectrum fungicide fludioxonil (100 µg/ml) (Zhao et al., 2018). Thus, AcuM and Pas2 are not required for environmental stress resistance in pathogenic fungi *A. fumigatus* and *C. neoformans*.

Overall, AcuK and AcuM homologs appear to regulate fungal responses to antifungal drugs and cell wall stress in some fungal species.

### Control of iron metabolism

As mentioned previously, studies on the opportunistic fungal pathogen *A. fumigatus* have revealed an unexpected role for AcuK and AcuM in regulating iron metabolism. AcuM was initially identified as a virulence regulator because the Δ*acuM* mutant exhibited significantly reduced virulence in a screen of *A. fumigatus* transcription factor deletion mutants using an animal infection model (Liu et al., 2010). Also, the deletion of AcuK resulted in attenuated virulence in the murine infection model, although the level of attenuation was lower compared to that observed in the Δ*acuM* mutant (Pongpom et al., 2015). In addition to its conserved role in controlling gluconeogenesis, transcriptional profiling suggests that AcuM also regulates genes involved in reductive and siderophore-mediated iron acquisition. The Δ*acuK* and Δ*acuM* mutants display impaired iron uptake, reduced extracellular siderophore production, and an inability to grow under iron-limiting conditions (Liu et al., 2010; Pongpom et al., 2015). Consistent with another study, *A. fumigatus* Δ*acuK* and Δ*acuM* mutants in the A1160+ strain background also exhibited decreased siderophore production (Caballero et al., 2024). However, this study suggested that the observed siderophore defects were due to metabolic dysregulation rather than direct transcriptional control of genes involved in iron acquisition (Caballero et al., 2024).

In *T. marneffei*, Δ*acuK* and Δ*acuM* mutants exhibited severe growth defects under iron-limiting conditions, indicating that AcuK and AcuM regulate iron metabolism (Wangsanut et al., 2024; Amsri et al., 2021). However, unlike *A. fumigatus ΔacuK* and *ΔacuM* mutants, the *T. marneffei* mutants maintained normal siderophore production despite losing AcuK and AcuM functions. Paradoxically, genes involved in reductive iron acquisition and siderophore biosynthesis were upregulated in Δ*acuK* and Δ*acuM* mutants (Amsri et al., 2021; Wangsanut et al., 2024; Liu et al., 2010; Pongpom et al., 2015). Transcriptional profiling further suggested that AcuK and AcuM primarily activate genes involved in iron utilization pathways, including iron-sulfur cluster proteins and components of oxidative phosphorylation (Wangsanut et al., 2024). The distinct patterns of AcuK and AcuM in regulating iron metabolism in the dimorphic fungus *T. marneffei* suggested the functional evolution of these transcription factors.

In *C. albicans*, the roles of Cwt1 and Zcf11 in iron metabolism have not been directly investigated. However, genome-wide ChIP-chip analysis revealed that Cwt1 binds to the promoters of *FET34* and *ISA1*, encoding a multicopper oxidase and an iron-sulfur cluster protein, respectively (Sellam et al., 2012). Additionally, transcriptomic analysis demonstrated that proper expression of *FET34* and the high-affinity iron permease *FTR1* is Cwt1-dependent, particularly under nitrosative stress conditions (Sellam et al., 2012). In *C. neoformans*, the roles of Pas2 and Rds2 in iron homeostasis remain uncharacterized. Nonetheless, transcriptomic analyses indicate that *CaFTR1* (CNAG_06242), which encodes a high-affinity iron permease, is downregulated in the *pas2* deletion mutant (Zhao and Lin, 2021).

Notably, AcuK and AcuM do not appear to regulate iron metabolism in the non-pathogenic filamentous fungus *A. nidulans* (Liu et al., 2010). The divergence in iron regulatory functions between *A. nidulans* and *A. fumigatus* suggests that AcuK and AcuM have undergone transcriptional rewiring in pathogenic fungi to support traits essential for virulence, such as iron homeostasis (Pongpom et al., 2015; Liu et al., 2010). These findings underscore the evolutionary adaptation of AcuK and AcuM homologs in fungal pathogens, indicating their role in pathogenicity and host adaptation.

Overall, these findings suggest that different fungal pathogens—and even different species of the same genus—employ AcuK and AcuM homologs in distinct ways to regulate iron-dependent pathways.

### Control of hypoxic response

Oxygen is required by fungi, as it is used in the biosynthesis of sterols, heme, and NAD +. It also serves as the terminal electron acceptor in aerobic respiration, making it essential for the generation of chemical energy, ATP (Puerner et al., 2023). At the site of infection, oxygen can become limiting, often dropping below 5% due to consumption by both fungal and host cells (Puerner et al., 2023). Under low-oxygen conditions, cells undergo metabolic reprogramming to enhance glucose flux through glycolysis while reducing reliance on oxygen-dependent pathways, such as the tricarboxylic acid (TCA) cycle and oxidative phosphorylation. Importantly, the Pas2 of *C. neoformans* has been identified as a key regulator of hypoxic adaptation, as the *Δpas2* mutant exhibited severely impaired growth under cobalt chloride-induced hypoxia and in a hypoxic chamber (Zhao and Lin, 2021). Co-immunoprecipitation (Co-IP) coupled with mass spectrometry revealed that Pas2 physically interacts with Rds2, and therefore, the Pas2-Rds2 complex functions as a regulatory unit in hypoxic adaptation. In response to low oxygen levels, the Pas2 complex regulates metabolic rewiring between respiration and fermentation. Notably, the roles of Pas2 and Rds2 in hypoxia are functionally independent of their roles in alternative carbon metabolism (Zhao and Lin, 2021).

Interestingly, hypoxia is well documented to increase NO levels (Poyton et al., 2009). As discussed earlier in the control of nitrosative stress response section, *C. neoformans* induces the *YHB1* gene under hypoxic conditions (**Table 2**), and this induction is dependent on Pas2, as it is abolished in the *Δpas2* mutant (Zhao and Lin, 2021). Thus, in addition to metabolic adaptation, we speculated that the Pas2/Rds2 transcription factors regulate the expression of nitric oxide dioxygenase gene to detoxify nitrosative stress under hypoxic conditions, providing another crucial adaptation mechanism in these environments.

The sterol regulatory element-binding protein (SREBP) pathway is a well-characterized regulator of hypoxic growth in fungi, primarily controlling ergosterol and lipid biosynthesis (Chang et al., 2007; Bien and Espenshade, 2010). However, epistasis analysis demonstrated that the Pas2 complex regulates hypoxic adaptation independently of the SREBP pathway. Multi-omics analyses further support this distinction, as SREBP does not appear to drive glycolytic gene upregulation or mitochondrial oxidative phosphorylation downregulation under hypoxia (Barker et al., 2012; Chang et al., 2007; Chun et al., 2007; Todd et al., 2006; Lee et al., 2007). Thus, Pas2 and Rds2 function differently from the SREBP regulator, as the Pas2-Rds2 complex specifically regulates metabolic reprogramming in response to hypoxia in *C. neoformans*.

Based on the evidence presented here, the Pas2-Rds2 complex regulates metabolic adaptation and likely the nitrosative stress response under hypoxic conditions in *C. neoformans*. However, the direct role of other AcuK/AcuM homologs in hypoxic responses, particularly during host microbial infection, remains unexplored.

### Control of protein translation

In *C. albicans*, the deletion of *CWT1* leads to altered expression of genes involved in ribosome biogenesis and protein synthesis, suggesting a role for Cwt1 in translational regulation (Moreno et al., 2007). Similarly, in *T. marneffei*, transcriptomic analysis of *ΔacuK* and *ΔacuM* mutants revealed significant differential expression of genes associated with ribosome biogenesis and protein synthesis (Wangsanut et al., 2024). These findings indicate that, in addition to their established roles in metabolic regulation and stress responses, AcuK and AcuM homologs may also regulate protein translation in specific fungal species. Further studies are needed to clarify whether this function is conserved across different fungal lineages.

## Mechanisms of the AcuK and AcuM proteins in regulating target gene expression

This section examines the mechanisms by which AcuK and AcuM regulate their target pathways, providing insights into the diverse array of genes controlled by these transcription factors. Two essential components for AcuK and AcuM function are their DNA-binding capabilities and their protein-protein interaction networks.

### DNA recognition and binding

Zn_2_Cys_6_ transcription factors possess a characteristic cysteine-rich DNA-binding domain that enables them to recognize and bind specific DNA motifs (**Figures 2B, C**). These proteins preferentially bind CGG triplet repeats, which can be arranged in inverted (CGGN_X_CCG), direct (CGGN_X_CGG), or everted (CCGN_X_CGG) orientations (MacPherson et al., 2006). In *A. nidulans*, AcuK and AcuM specifically recognize CCGN_7_CCG motifs, where two CGG triplets are separated by seven nucleotides (Suzuki et al., 2012) (**Table 3**). The CCGN_7_CCG motif is highly conserved across *Aspergillus* species and *T. marneffei*, where it is found in the promoters of numerous genes involved in gluconeogenesis, reinforcing the essential role of AcuK and AcuM in carbon metabolic reprogramming (Suzuki et al., 2012; Wangsanut et al., 2024).

Mutational analysis in *S. cerevisiae* has revealed a novel mode of DNA binding for the Gsm1/Rds2 and Ert1/Rds2 complexes (Martinez et al., 2024). Unlike traditional Zn_2_Cys_6_ regulators, these transcription factors do not bind adjacent half-sites but instead recognize short, common DNA stretches. For example, Gsm1 binds to CCGG motifs, a compacted form of CGG triplets arranged in overlapping, opposite orientations (**Table 3**). Additionally, Gsm1 and Rds2 cooperatively bind to the *FBP1* promoter at the sequence CCGGAGTTA, further highlighting the diversity of Zn_2_Cys_6_ protein binding mechanisms (Martinez et al., 2024).

As mentioned previously, *N. crassa*, Aod2 and Aod5 recognize AIM motif located in the *aod-1* promoter (Chae et al., 2007; Qi et al., 2017) (**Table 2**). The AIM consists of two directly repeated CGG triplets separated by seven base pairs, a structure consistent with the AcuK/AcuM binding motif found in gluconeogenic genes of *A. nidulans*. Similarly, in *C. albicans*, Cwt1 and Zcf11 bind to the *C. albicans* Antimycin A Responsive box (CARbox) region within the *AOX2* promoter (Liu et al., 2023). The CARbox contains three CGG triplets separated by either 31 or 22 base pairs, forming two CGGN_7_CGG-like motifs within the flanking sequence CGA(A/T)_7_CCGG(A-T-rich) CCGTCATATTCCA (**Table 3**).

For nitric oxide detoxification genes, AIM-like sequences have been identified within the promoter of flavohemoglobin genes in *N. crassa* (Qi et al., 2017). However, Cwt1 binding to the *YHB1* promoter in *C. albicans* follows a different mechanism. Instead of targeting CGG triplets, Cwt1 binds to the CAAGTTTAAAGACCGAGAGAATGA sequence, which lacks the general Zn_2_Cys_6_ CGG triplet signature (Sellam et al., 2012) (**Table 3**). This deviation suggests that Cwt1 may utilize an alternative DNA recognition mechanism for nitric oxide detoxification genes, further highlighting the functional diversity of AcuK and AcuM homologs across fungal species.

### Protein-protein interaction

Heterodimerization of AcuK and AcuM is essential for their transcriptional regulatory function. While the specific domains required for AcuK-AcuM protein interactions have not been fully characterized, a conserved PAS; **Figures 2A, B**) is hypothesized to mediate their complex formation. PAS domains are widely recognized for their involvement in eukaryotic signal transduction and dimerization events, suggesting a functional role in AcuK-AcuM interactions (Taylor and Zhulin, 1999; Xing et al., 2023).

In *A. nidulans*, PAS domain mutations impair AcuK/AcuM function, reinforcing the domain’s importance in transcriptional regulation. A double mutant carrying alanine substitutions in the PAS domain (*acuM^G418A^ acuK^G535A^*) exhibited enhanced sensitivity to antimycin A and severe growth defects on gluconeogenic carbon sources, phenotypes that were more pronounced than those of single PAS mutants (*acuM^G418A^* or *acuK^G535A^*, **Figure 2A**) (Suzuki et al., 2012). Additionally, an *A. nidulans* strain carrying a chimeric fusion of *acuM* (lacking its PAS domain) with the PAS domain from *S. cerevisiae RDS2* retained full functionality in alternative carbon metabolism (Suzuki et al., 2012). However, the AcuM-Rds2 fusion protein failed to function in the absence of *acuK*, providing strong evidence for a functional interaction between AcuK and AcuM via PAS domain sequences (Suzuki et al., 2012).

In *N. crassa*, point mutations in the PAS domain of *aod-2* and *aod-5* (*aod-2^G416A^* and *aod-5^G497A^*) resulted in hypersensitivity to antimycin A, further supporting the critical role of PAS residues in Aod2/Aod5 function, **Figure 2A** (Chae et al., 2007). Similarly, in *P. anserina*, mutations in the PAS domain of *rse2* (Δ*rse2^452^* and Δ*rse2^454-504^*) led to complete loss of growth in the presence of antimycin A, indicating that Rse2 function is dependent on PAS domain integrity (Bovier et al., 2014).

**Figure 3:**
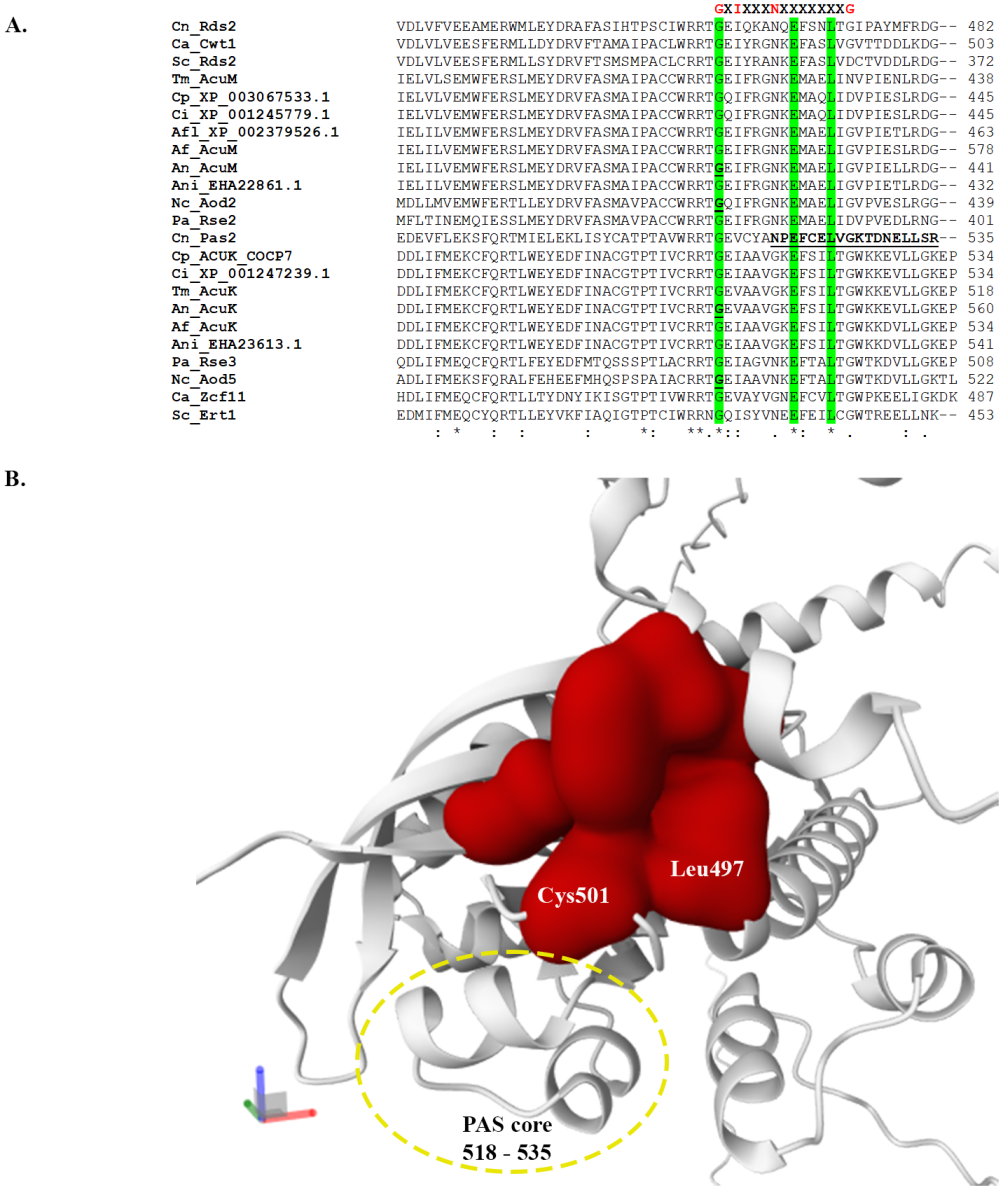
Computational analysis predicts the binding pocket in the PAS domain of the Pas2 protein from the human pathogen *C*. *neoformans*. **(A)** Protein sequences of the PAS domain from the AcuK and AcuM homologs were aligned using Clustal Omega. Top indicates the conserved consensus protein sequences for the PAS domain. Highlight in green illustrates residues that are conserved in AcuK and AcuM homologs from all examined fungal species. Underlined depicts residues that mutagenesis studies have experimentally shown to be important for protein functions. **(B)** Protein’s ligand binding sites were predicted using the PrankWeb (https://prankweb.cz/). The input protein structure for the Pas2 protein from *C*. *neoformans* was modeled using the AlphaFold3 server, with the addition of two Zinc ions. The surface of residues for the predicted pocket is shown in red. Residues that are part of the PAS domains were indicated. PAS core is indicated by a yellow circle, including residues 518 – 535 (NPEFCELVGKTDNELLSR as highlighted in A.

In *C. neoformans*, the direct involvement of PAS domains in the interactions between Pas2 and Rds2 proteins has not yet been examined. However, the PAS domain function has been implicated in hypoxic adaptation, a key regulatory role of Pas2 (Zhao and Lin, 2021). A *C. neoformans* mutant expressing Pas2 with a deleted core PAS domain (Δ*pas2core*) was unable to grow under hypoxic conditions (**Figure 3**). Additionally, mCherry-tagged ΔPas2core protein mislocalized to vacuoles instead of the nucleus, suggesting that the PAS domain is essential for nuclear localization and/or protein stability, both of which are required for hypoxia adaptation (Zhao and Lin, 2021).

Collectively, these findings highlight the critical role of the PAS domain in AcuK and AcuM function, likely through mediating protein-protein interactions necessary for transcriptional regulation. Further structural and biochemical studies are needed to elucidate the precise mechanisms by which PAS domains facilitate heterodimerization and target gene activation.

## Role of AcuK and AcuM in response to combined stress

Oftentimes, fungi simultaneously encounter multiple hostile microenvironments, however, the regulatory responses to combined stresses remain largely unexplored in most human fungal pathogens. A recent study in *A. fumigatus* demonstrated that oxidative stress responses are highly dependent on nutrient availability (Emri et al., 2024). In this study, transcriptomic analyses were conducted under four glucose/iron conditions (rich or limited for each). The results revealed that iron metabolism plays a critical role in the fungal response to both iron limitation and oxidative stress. Specifically, siderophore secretion and the production of iron-sulfur cluster proteins were found to be essential for adapting to oxidative stress. Furthermore, distinct gene expression patterns were observed under different nutrient conditions, indicating that transcriptional regulators play a crucial role in governing fungal adaptation to combined stressors. Given the involvement of AcuK/AcuM proteins in multiple metabolic pathways and stress responses, we propose that AcuK and AcuM serve as key regulators of fungal responses to combined stress. To support this hypothesis, we reviewed the literature and presented the evidence below.

The role of AcuK and AcuM in coordinating responses to iron and carbon (glucose) limitation was directly tested in *A. fumigatus* (Caballero et al., 2024). In the A1160+ strain background, *ΔacuK* and *ΔacuM* mutants exhibited growth defects when glucose was used as a carbon source and glutamine or ammonium (NH_4_^+^) as nitrogen sources, regardless of iron availability. These findings suggest that AcuK and AcuM broadly regulate carbon metabolism beyond gluconeogenesis, rather than acting as direct regulators of iron homeostasis. Gene expression analyses further indicated that iron limitation influences carbon metabolism in both AcuK/AcuM-dependent and independent manners. For instance, *acuK* and *acuM* were strictly required for the transcription of *acuF* and *acuD* (encoding isocitrate lyase of the glyoxylate cycle), independently of iron levels. In contrast, *acuG* expression depended on AcuK and AcuM only under iron-sufficient conditions. Additionally, AcuK and AcuM were essential for the expression of *acuN* under iron-limited conditions, but not when iron was abundant. Notably, *acuE* (encoding malate synthase of the glyoxylate cycle) was upregulated during iron starvation, with its expression partially dependent on AcuK and AcuM. These findings suggest that AcuK and AcuM coordinate carbon metabolism in response to iron levels, likely fine-tuning metabolic flexibility in response to fluctuations in nutrient availability.

Several studies provide indirect evidence, based on experimental data, that AcuK and AcuM also mediate responses to integrated signals arising from respiratory inhibitors and nutrient stress. In *C. albicans*, the hypersensitivity of *Δcwt1* and *Δzcf11* mutants to antimycin A was dependent on the type of available carbon source; both mutants exhibited growth defects only when grown on non-fermentable carbon sources (Liu et al., 2023). Similarly, in *N. crassa*, Qi et al., 2017 proposed that different environmental signals influence transcriptional regulation in an *aod2*- and *aod5*-dependent manner (Qi et al., 2017). For example, different levels of chloramphenicol-induced AOX gene expression were observed when *aod2* and *aod5* mutants were grown under different nutrient conditions (Qi et al., 2017). Additionally, *pepck* gene expression levels differed significantly between nitrogen-limited and nitrogen-rich (Vogel’s medium) conditions in the presence of chloramphenicol. These findings suggest that AcuK and AcuM proteins integrate transcriptional regulation of gluconeogenesis and alternative respiration in response to both nutrient availability and respiratory stress.

Additional support for AcuK/AcuM’s role in combined stress responses comes from comparisons between AOX-deficient mutants and Δ*acuK/*Δ*acuM* mutants. Specifically, fungi lacking alternative oxidase genes maintain normal responses to glucose limitation and environmental stress, whereas Δ*acuK* and Δ*acuM* mutants exhibit pleiotropic defects across multiple fungal species (**Table 2**). For example, in *A. nidulans*, the Δ*aodA* mutant grows normally on gluconeogenic carbon sources such as acetate and proline (Suzuki et al., 2012). Moreover, the induction of *acuF* by acetate and proline is not impaired in the Δ*aodA* mutant, indicating that AOX is dispensable for gluconeogenesis and metabolic adaptation to glucose limitation. Similarly, *A. nidulans* Δ*aodA* mutants do not display increased sensitivity to oxidative stressors such as hydrogen peroxide or menadione. In *C. albicans*, AOX-deficient mutants (Δ*aox2* and Δ*aox1*Δ*aox2*) also grow normally under alternative carbon sources and show no increased sensitivity to cell wall stressors (calcofluor white, Congo red) or altered macrophage recognition (Liu et al., 2023; Duvenage et al., 2019). In contrast, mutations in *acuK* and *acuM* often result in severe growth defects and dysregulation of multiple pathways, underscoring their role as global regulators of metabolic adaptation and stress response.

Collectively, these findings highlight the function of AcuK and AcuM proteins as integrators of multiple environmental signals, enabling fungi to coordinate transcriptional responses to combined stress conditions. However, additional studies—such as those conducted by Caballero et al. (2024) (Caballero et al., 2024) and Emri et al (Emri et al., 2024)—are needed to elucidate further the molecular mechanisms underlying AcuK/AcuM-mediated regulation of combined stress responses.

## Signal integration and regulatory crosstalk by AcuK and AcuM

Although it has become clear that fungi often live in fluctuating environments and must respond to combined stress rather than individual stressors, and that AcuK and AcuM are involved in regulating many pathways, little is known about how AcuK and AcuM integrate diverse environmental inputs to enable growth, survival, and fungal virulence. One of the key structural features of AcuK and AcuM is the presence of dimerization and PAS domains (**Figures 2**, **3**). These functional domains likely facilitate the formation of protein complexes, enabling AcuK and AcuM to interact with diverse array of protein partners and thereby co-regulate multiple signaling and metabolic pathways. A distinct protein-protein interaction network may enable AcuK and AcuM to activate different sets of genes through variations in promoter binding. For example, in *A. nidulans*, AcuK-AcuM heterodimer is known to regulate genes associated with gluconeogenesis (Suzuki et al., 2012), whereas in *A. fumigatus*, a homodimer of AcuK or AcuM is involved in the regulation of iron metabolism (Liu et al., 2010; Pongpom et al., 2015).

In *C. neoformans*, a Co-IP assay coupled with mass spectrometry has identified the regulator of iron homeostasis Cir1 and the global stress transcription factor Atf1 as interacting partners of Pas2, in addition to Rds2 (Zhao and Lin, 2021). In fact, Cir1 is the homolog of SreA (Jung et al., 2021), a GATA-type negative regulator of siderophore biosynthesis and iron homeostasis that is highly conserved in *N. crassa*, *Aspergilli*, and *T. marneffei* (Zhou et al., 1998; Haas et al., 1999; Schrettl et al., 2008; Amsri et al., 2022; Pijuan et al., 2023). Pas2 was detected to directly interact with Cir1 under both normoxic and hypoxic conditions. In *A. fumigatus*, AcuM has been found to activate siderophore production, most likely through the repression of *sreA* gene, as indicated by the transcriptomic study (Liu et al., 2010). Notably, the *A. funmigatus* SrbA (SREBP homolog, a regulator of hypoxia) also mediates the regulation of iron acquisition in response to hypoxia and iron limitation by coordinating the expression of genes involved in both iron uptake and ergosterol biosynthesis (Blatzer et al., 2011). Thus, a model linking the transcription regulators AcuM, SreA, HapX, and SrbA in coordinating gluconeogenesis, iron acquisition, and ergosterol biosynthesis has been proposed (Blatzer et al., 2011). Aside from the study in *C. neoformans*, a direct Co-IP assay has not been performed to detect protein-protein interaction between AcuK/AcuM with SreA in *A. fumigatus* or other fungal pathogens. Based on the studies in *C. neoformans* and *A. fumigatus*, Pas2 and AcuM can regulate iron-dependent pathways either by directly interacting with the SreA protein (Zhao and Lin, 2021) or by suppressing the expression of the *sreA* gene (Liu et al., 2010), respectively.

In addition, Atf1 is the homolog of AtfA, a bZIP-type regulator of the ATF/CREB (activating transcription factor/cAMP-responsive element-binding) protein family that plays an important role in stress response and development in many fungi, including *N. crassa*, *Aspergilli*, and *T. marneffei* (Leiter et al., 2021). The Pas2-Atf1 interaction was found only under hypoxic conditions. This result suggests that Pas2/Rds2 can form a protein complex with AtfA to integrate stimuli ranging from low oxygen availability to various environmental stressors.

In *C. albicans*, the Cwt1/Zcf11 proteins are part of multifactor complexes that control *AOX2* transcription in response to distinct environmental stressors, including respiration inhibitors, reactive oxygen, nitrogen, and sulfur species, as well as copper starvation. The *AOX2* transcriptional network comprises Cwt1/Zcf11, the bHLH factors Rtg1/Rtg3, and Zcf2 regulators (Liu et al., 2023). No inter-complex interaction was found to form a super-complex between Cwt1/Zcf11 and Rtg1/Rtg3. However, the highest transactivation of the *AOX2* gene expression requires the binding of two complexes. Thus, the heterodimers of Cwt1/Zcf1 and Rtg1/Rtg3 cooperatively bind the CARbox to achieve maximal *AOX2* transactivation. While a single gene deletion mutant (*RTG3*, *CWT1*, and *ZCF2*) showed only specific stress response impairment, a triple gene deletion mutant was defective in activating the *AOX2* gene expression in response to all tested stressors (antimycin A, KCN, menadione, sodium sulfite, sodium nitroprusside, DPTA NONOate). This genetic analysis supported a model in which the integration of distinct environmental stress signals could be achieved through the cooperative binding of Cwt1/Zcf1 with other transcription factors at the CARbox of the *AOX2* promoter.

With valuable insights from studies of *C. neoformans* and *C. albicans*, further experimental validation is essential to establish the generalizability of this regulatory crosstalk model to other human fungal pathogens. Comprehensive investigations using advanced proteomic approaches, such as Co-IP coupled with mass spectrometry or ChIP-seq, hold great promise for identifying novel protein partners and direct target genes of AcuK and AcuM. These strategies will not only deepen our understanding of the intricate regulatory networks involving AcuK and AcuM, but may also uncover new facets of their roles in fungal metabolism and virulence.

## Exploiting AcuK and AcuM as antifungal drug targets

Since AcuK and AcuM coordinate the expression of multiple genes in response to glucose, iron, and oxygen limitations, as well as respiratory and environmental stresses, pharmacological inhibition of AcuK and AcuM function could simultaneously disrupt multiple virulence and fitness-related traits in fungal pathogens, providing a promising avenue for antifungal intervention. Recent breakthroughs have demonstrated that transcription factors can serve as promising drug targets, with small molecules effectively inhibiting their activity (Xu et al., 2019; Yuan et al., 2024; Scheuermann et al., 2013). For instance, Belzutifan (PT2977; MK-6482), a HIF-2α inhibitor, has been approved by the FDA for the treatment of von Hippel-Lindau disease (Curry and Soleimani, 2024). Belzutifan binds to the PAS-B domain of HIF-2α, inducing conformational changes that prevent heterodimerization of the HIF complex, thereby impairing its ability to bind DNA. Given that PAS domains often function as environmental sensors, their regulatory activity is frequently modulated by ligand-induced allosteric changes that influence protein-protein interactions (Harper et al., 2003). This evidence presents a unique opportunity to exploit PAS domains as selective targets for small-molecule inhibitors. Given that PAS domains have been successfully targeted in therapeutic applications, and AcuK and AcuM proteins contain PAS domains, a similar approach could be explored for AcuK and AcuM homologs.

To facilitate structure-guided drug design, structural studies using crystallography and cryo-EM will be essential for elucidating the three-dimensional architecture of AcuK and AcuM, particularly their PAS and Zn_2_Cys_6_ domains. To explore the feasibility of structure-based drug design, we selected Pas2 for molecular modelling, as it is the only AcuK/AcuM homolog in human fungal pathogens whose PAS domain has been experimentally validated as essential for hypoxic adaptation (Zhao and Lin, 2021). In silico structural analysis (**Figure 3**) reveals that several PAS domain residues form a distinct binding pocket in the Pas2 protein, highlighting an exciting potential for ligand-mediated allosteric regulation (Jakubec et al., 2022). In addition to the PAS domain, the Zn_2_Cys_6_ DNA-binding domain represents another promising target for antifungal drugs, as it is fungus-specific and completely absent in human host proteins. The unique structural features of both PAS and Zn_2_Cys_6_ domains in AcuK and AcuM transcription factors position them as attractive targets for small-molecule inhibitors.

Future directions in fungal infection therapy may focus on combination strategies that exploit the synergy between transcription factor inhibitors, such as PAS inhibitors or anti-AcuK/AcuM antibodies, and established antifungal agents, including azoles or echinocandins. By targeting fungal adaptation mechanisms, transcription factor inhibitors can suppress stress response pathways and adaptive transcriptional changes, rendering fungi more vulnerable to traditional drugs. This dual approach not only disrupts essential processes, such as ergosterol synthesis and cell wall integrity, but also reduces the likelihood of resistance development by limiting the activation of drug efflux pumps and mutations in drug targets. Additionally, these combinations may enable lower drug dosages, thus minimizing toxicity while enhancing efficacy. Such innovative therapeutic strategies hold great promise in overcoming resistance and improving treatment outcomes, particularly for invasive fungal infections caused by opportunistic fungi, including *A. fumigatus, T. marneffei, C. neoformans*, and *C. albicans*.

In addition to structural studies, several key research areas should be prioritized. CRISPR-based functional genomics, genome-wide ChIP-seq and transcriptomic analyses are necessary to map direct AcuK/AcuM target genes, providing insights into their role in metabolic rewiring and virulence regulation. Further biochemical and biophysical studies should investigate how AcuK and AcuM dimerization and interactions with co-regulators influence transcriptional activity under conditions of nutrient limitation, hypoxia, and oxidative stress. By addressing these research gaps, future studies will provide critical knowledge for developing novel antifungal strategies that target fungal adaptation and virulence.

## Artificial intelligence methodologies to characterize AcuK and AcuM

Emerging AI methodologies can substantially accelerate the dissection and therapeutic exploitation of AcuK/AcuM networks. First, AI-driven drug discovery approaches have become increasingly valuable (Ocana et al., 2025). The AI-powered AlphaFold tool, which supports protein structure prediction (Desai et al., 2024), enables virtual screening and molecular docking to identify small molecules that can inhibit the activity of transcription factors (Lin et al., 2025). In parallel, ligand-based drug design and AI-driven platforms can generate and prioritize novel compounds even in the absence of resolved transcription factor structures. Systems biology and network pharmacology approaches further facilitate the identification of key regulatory nodes within fungal virulence networks, guiding rational drug targeting (Li et al., 2025). Moreover, early-stage *in silico* screening for toxicity and pharmacokinetic properties helps streamline the drug development pipelines. These computational strategies, when integrated with experimental validation, form a powerful, cost-effective framework for developing next-generation antifungal therapeutics targeting virulence-associated transcription factors.

Second, sequence-to-function models (convolutional and transformer architectures) trained on promoter regions of validated targets can learn the “motif grammar” of AcuK/AcuM (CGG-based repeats with variable spacing and orientation), enabling genome-wide prediction of binding sites and cross-species transfer learning (Alipanahi et al., 2015; Hu et al., 2023). Third, multitask predictors that integrate transcription factor occupancy with pathway topology can forecast the expression of sentinel genes (e.g., *acuF*/*acuG*, *aox*, *YHB1*/*FHB1*) under combined glucose, iron, hypoxia, and nitrosative challenges, thereby quantifying AcuK/AcuM’s role as an integrator of convergent stresses. Moreover, causal graph learning applied to time-series perturbation data (Δ*acuK*/Δ*acuM* and environmental shifts) can resolve directionality within the regulatory network while using ChIP constraints as priors, yielding a compact, testable set of edges for CRISPR or reporter validation.

For therapeutic translation, AI-accelerated chemistry offers a tractable route to small-molecule modulators. Pocket-conditioned generative models and machine learning-based docking/scoring can prioritize ligands targeting the PAS pocket or interfering with Zn_2_Cys_6_-DNA engagement, followed by biophysical and transcriptional assays and synergy testing with standard antifungals. Complementarily, a “digital twin” of fungal metabolism, combining flux balance analysis with reinforcement learning, can propose intervention combinations that collapse stress adaptation while preserving selectivity. Finally, Large Language Model (LLM)-assisted curation can maintain a living AcuK/AcuM knowledge graph (genes–motifs–conditions–phenotypes) to standardize nomenclature across species and continuously update candidate targets, tables, and figures. Together, these AI-enabled strategies provide an end-to-end framework—from sequence motifs and stress-condition prediction to lead discovery and network-level intervention design—that will sharpen hypotheses and shorten experimental cycles.

## Conclusion and perspective

Molecular genetic studies coupled with multi–omics analyses have shown that loss of AcuK and AcuM proteins leads to pleiotropic effects on both phenotype and global gene expression, strongly reinforcing their roles as master regulators of cellular adaptation to combined stresses across multiple fungal species. Their most highly conserved target genes are involved in gluconeogenesis, such as fructose-1,6-bisphosphatase and phosphoenolpyruvate carboxykinase, and alternative respiration, particularly the alternative oxidase gene. Another key conserved target is the flavohemoglobin/nitric oxide dioxygenase, a crucial enzyme for reactive nitrogen species detoxification that operates in an oxygen- and heme-dependent manner. Notably, the contribution of AcuK and AcuM to iron homeostasis appears to be unique to fungal pathogens, where they regulate the expression of iron metabolic genes through both direct control of iron- and siderophore-uptake genes and indirect modulation of iron-dependent pathways. Beyond these functions, AcuK and AcuM homologs have been implicated in cell wall integrity, drug resistance, hypoxic adaptation, and protein translation, underscoring their diverse and central roles in fungal physiology and pathogenicity.

AcuK and AcuM proteins represent promising targets for antifungal therapy, as they regulate both fungal fitness and virulence attributes (Brown et al., 2014). Structural analyses suggest that both the Zn_2_Cys_6_ DNA-binding domain and PAS domain are druggable, providing viable avenues for therapeutic intervention. First, the Zn_2_Cys_6_ domain is unique to fungi and has no homologs in human host proteins, making it an attractive target for fungus-specific drugs. Second, the PAS domain possesses structural features that can be allosterically exploited by small molecules, as exemplified by the successful development of Belzutifan, a PAS-B domain inhibitor targeting HIF-2α.

Future research should focus on elucidating the structure-function relationships of AcuK and AcuM proteins and conducting direct experimental validations under combined stress conditions. Incorporating AI-based drug discovery and AI-assisted functional genomics analyses will help unravel their complex regulatory mechanisms and accelerate the identification of small-molecule inhibitors that disrupt fungal adaptation and pathogenicity. Altogether, these approaches will pave the way for novel antifungal therapeutic strategies.
