# Supplementary material for: Regulatory functions of AcuK and AcuM transcription factors in fungal metabolic adaptation, stress response, and virulence
Source: Front Cell Infect Microbiol. 2025 Dec 8;15:1717070. doi: 10.3389/fcimb.2025.1717070 (PMC12719309; doi:10.3389/fcimb.2025.1717070)
Supplement: Supplementary file 1 [file Table1.docx]

**Supplemental information S1**

**Amino acid sequences of AcuK and AcuM homologs used in Phylogenetic analysis (Figure 1)**

**AcuK homologs**

>Cn_Pas2

MQDHQWVHPEGLSSAGSPLSFTSGTPTHDASLFNYAPSHHHHRPSTSSGYHMINPSGLGRSSSGIYVGGMGGLTTVAPTAIPPPVAVEKPLVPVFGGDSGRGRPKSKNPKAKPQGLGTERKARKKVAKACLACQKSHLTCDEQRPCTRCVKKGMADKCVEGVRKKAKYLMEGDERLSSSMQSQASSVHSMSPQSIHQSLPTMASLPSQSTDSQMQDVWLASALQRQNPTPNINIAPGINMNQGDVWGTQSAEITPGAYSGYSSTAGTAANNEYQMLDAMFSSLSPVFPGLNDSLDDASRQIANVAQAGTNLDLVNNSVSNSFDPQQPWINPSPSQSANTYNPTPSGSSVQGAGISPSAYGGSDVSPHQQWGWGSMDAPGQIMLDGSGAPGQQNQVDLGQVRGPGTVTALNEWRRTNKALTPQEVYRAVVRPYEYTQGYHFLMDYLTKNFETNDVLRVVRSLASFRPSLIALQMSMSEEDEVFLEKSFQRTMIELEKLISYCATPTAVWRRTGEVCYANPEFCELVGKTDNELLSRKTYIYQLFAKPSVVSYWEGFSTHAFENTTQNFFQSISLALNTGGVNPCTCCFTIRRDVFDLPSVVIGQFLPIPAETQ

>Ca_Zcf11

MKIKQENITNTTSINTSNVNDKPKPKRKKTSRACNHCHKAHMTCDSGRPCKRCIQRGLDSTCEDAPRKRKKYLQDVPNSSLMSNHSMNSSDNLDSNGVSPMQPTLSQPMPPVQESSLPTNIPNQSQVPSQSPNTASQKQLSYSPPLASTYSPNHKTYSATLFQHSATSPELMQTVPEYFPELYNQHYQPSANPNQKRRTNFLSTAADLEYSTLSNILQENFGHHTTSNEGTPNSHNFSPALSPHNMPTNNTNAPSTSTQLLTNDQQRQATSFNNHTKHNTPSPLNTSHTKLYEDARYPKCDETINQYFLGDTDSGKMVVFPDVLTAIENMKNNDPAVYLERNSKSALSFVMSIQHENNSNGGKEDQLFKEPEEIYEKVKKPFSYTPGYHSLIAYLRKRFTKPMLVKMAESMATYRPSFIACTNSLKEHDLIFMEQCFQRTLLTYDNYIKISGTPTIVWRRTGEVAYVGNEFCVLTGWPKEELIGKDKRKFIVELLDDKSVLQYFQVFSRIAFGDFLGATMTECTLLTPNPNVKIRTGCMWTLKRDVFGIPMMIVGNFLPIL

>Sc_Ert1

MCTPDENDYKTSTDPDTSANTNHTLEKKKRKKRKNTNVACVNCSRLHVSCEAKRPCLRCISKGLTATCVDAPRKKSKYLAGIPNRELPMNIQPDLPPRKIMIPIYNNSSNSSLNVNNMGEQQKFTSPQHIVHKAKFLSNAADSEYSILSNIIYQDTLSNKIPIDILYSNTNSTSNSTIGNSSNNSPTGTNTSPEETEMEKIRQLYSEQRANIPPHPYPSSNQNVYSILLGPNSAKIVASQVNLFANHFPLVPVDSADNSLNFKRLLPRDPSEKSSQINWDSSINQYYLNSETVTFPELAIPLKRRKNHLVSVSLESCSPDAANIKSNVEWEHSLRYSTPMEIYTSINAPFSHTPGFHHLLVYLKHRFNQQDLVKMCRSIAEFRPIFIACSVTLTEEDMIFMEQCYQRTLLEYVKFIAQIGTPTCIWRRNGQISYVNEEFEILCGWTREELLNKMTFIVEIMDDESVRDYFKTLSKVAYRDFRGSEKMKVCRLLSPIKGKIIHCCCMWTLKRDVSGLPLMILGNFMPILN

>Pa_Rse3

MPEDGGPFGSEAAEASGAMSETENEYDDHEPHHKDEDDRMSEQNTTPDGVDAGGEVKKKYDPKDPLRPRRKKARRACYACQRAHLTCGDERPCQRCIKRGLQDSCQDGVRKKAKYLHDAPPEALRPVLGPNYNPNAPSSRHGGQRHHSVSTDASTVRTFFSHSNASQYPVYSSTQSIPHGLTESLPFNSQQSPVSPTFQQTSSNPPISGMVAPPVSSPMTPFGLPFDPSDPNIFNFNIDGLNFGSHYGAMEFGMLGHMSSSAADTPPQESGMGQQPGDVHFGAGLFGSHFDNRMLPEFLGLDAGANGIYSQGNLQHGLPHAYAIPAGPTSLQSPSTENNSPQPTTFGFDDRPSPTMSQYPNAPGAKSSSNSRPSKLRKLDKVAILQKRQRDPSYIYDTVKKSFDYVGSFHKLFEVLSSRFSQPHAARIAKSLAAIRPALLASTRNLTTQDLIFMEQCFQRTLFEYEDFMTQSSSPTLACRRTGEIAGVNKEFTALTGWTKDVLLGKEPNRNTNLGGTGVRTTPRLKSLNESSAENGGAASGPRPVFLAELMDHESAVEFYEDYSQLAFGDSRGRMTRKCRLLKYRTDKPAAGGGGGAGEEERKPDPSAAPRQQEKDSRHSILSNRVAKIDGEHGISKLERDGKLECSYTWTIKRDMFDMPMLFVINVRFFFFFDDYYGRRHC

>Nc_Aod5

MPDDVGPAEAEVSGAVSESDNEYDETEVTTKDDDDEKMAERSVASEGVETNGDQKKKYDPKDPLRPRRKKARRACYACQRAHLTCGDERPCQRCIKRGLAEACQDGVRKKAKYLHDAPPEALRPVLGPNYNPAAAVSVRNGHRHPSNAGSDAGSSIGTFYSQSTQYPVFSSAATQLGSIPENLPFPQQSPVSPTFQPSSNPQLGSIGVSSVSSPMNSFPPALFDPSNPAIFNFNLEGLNFGSQYGAMEFGMLGHMSSGAAETPPRDPSMAQQGTSDVGFNPSGVFGNGLNQFEKVYDNNTGLISDFLTLDAHSNGLYSQGNLQHGLPHAYAIPAGPTSLQSPSTENNSPQPTGFGFESPTATNYTGVPGAAGNQPGSQQPRAQKPKTPALGKLGPQSVLGKRQRDPSSIYEAVKEPFQYVASFHKLISLLQNRFSGASTISIVRSLASIRPSFMSCMKTLNRADLIFMEKSFQRALFEHEEFMHQSPSPAIACRRTGEIAAVNKEFTALTGWTKDVLLGKTLNLNANMGGTNSDTLSISSKGGRGGIVGTTPRLKPLHPEQGTNADSQQQQSQQHKEQPQPVFLAELMDEASVTQFYEDYAQLAFTHSRGTVVRKCRLLKYRTQENMDAAAAAAAAASAPTASGGSGSSNGTVVNGGPDSSPAGKTEKERPTGVNVASNSILSNRVAKIDGEHGISKLERDGKLECSYTWTIKRDVFDIPMIIMINFLPCYYRSHNQLAV

>Tm_AcuK

MMDTDKDDLPSATDHSEHESGDAVKVEGGASKTASNSKDPSRPRRKKARRACFACQRAHLTCGDERPCQRCIKRGLQDACHDGVRKKAKYLHDAPDGALVPGAQGNFYNQANGLPKISPTEYTQNGTNNAQQQQQKSGTIYASSTPSYNNNNGTFDTNNATNTVLPDSAALNPGPFTATPASPTFSISTSSAMPSLTQPTNDMTSGAGQGSFGAFFDPSDPALFNFDLASMNFGNRYGALEFGMLGHMATGAGDTPPSDNGNQRGGSVGQRSNSQQFGNPPGAFTTESPSQQSFMFGDPVLNDWSGGQNTNPRMNVGSGLYGQGGGGGGGMHLLQQDAPHAYAIGSNTFASPSSTTSPHATTIAPSQFDDSPMKTKTVISTPHLRQQSLYNSNSNKRRHREPSAIYDSVKEPYSYTGGFHKLIAFIKRRFSPPKTVRIAKALASIRPSFIATTKTLNQDDLIFMEKCFQRTLWEYEDFINACGTPTIVCRRTGEVAAVGKEFSILTGWKKEVLLGKEPNLNVNTGGGNNTSSQSDSTSSSIRGGAGGRMRNQEPGPNNMAPVFLAELLDDDSVIEFYEDFARLAFGDSRGSVMNTCKLLKYKTKEDMDSVNADDSRWNSAMTHHIVGKGGIVGEAGMNRLGFKDGRVECSYCWTVKRDVFDIPMLIVMNFLPHI

>Cp_ACUK_COCP7

MTANAINGPVLPTPLATPGDNNKSADTTMADQGTRPESQPQGQNNGAKPQNGQTKPMSAANAKDPLRPRRKKAKRACFACQRAHLTCGDERPCQRCIKRGIQNACHDGVRKKAKYLHDAPNEALMPHLQGHLYTTQANTARNTIPLTRNGSNSKTNFYPQQQSSFNNFYQNKPDPTLNQPQLHDTGRPDTFPSQSPVSPTFNMTANPAASGNQGLPSSLSASNSNASGQAQNGFGSAFFDPSDPALFNFDLASMNFGNHYGALEFGMLGHMATGAGETPPSDGATQHGSVGRSGSGTYTAGSNFGESPTGQPSFLFGDPTIGGDWTSSVNTRNIYGQNMNNMSETPHAFAIESAPANFASPNSIESPLLTNTTTFDDNTAPTYANRANINSVPSQRQPVVSTPQLKHLQVGKRRQRNPSAIYDSVKEPYSYTTGFHSLTAFIQRRFSPQNTLRIAKALASIRPSFIATTKTLNRDDLIFMEKCFQRTLWEYEDFINACGTPTIVCRRTGEIAAVGKEFSILTGWKKEVLLGKEPNLNINTGGSSGAMSGVTSRGSFTPRTGMDINPTGRTQPVFLAELLDDESVVEFYEDFAKLAFGDSRGSVMTTCKLLKYKTKADMDMLSGTTSSAGGENEHGAAGNGDVKPENGMGASNGQSQHSLQRQRKWSRGGIAGEAGMNQLGFKDGKVECSYCWTVKRDVFDIPMLIVMNFLPCI

>Ci_XP_001247239.1

MTTNAINGPVLPTPLATPGDNNKPADTTMADQGTRPESQPQGQNNGAKPQNGQTKPMSAANAKDPLRPRRKKAKRACFACQRAHLTCGDERPCQRCIKRGIQNACHDGVRKKAKYLHDAPNEALMPHLQGHLYTTQANTARHTMPLTRNGSNSKTNFYPQQQSSFNNFYQNKPDPTLNQPQVHDTGRPDTFPSQSPVSPTFNMTANPAASGNQGLPSSLSASNSNASGQAQNGFGSAFFDPSDPALFNFDLASMNFGNHYGALEFGMLGHMATGAGETPPSDGATQHGSVGRSGSGTYTAGSNFGESPTGQPSFLFGDPTIGGDWTSSVNTRNIYGQNMNNMSETPHAFAIESAPANFASPNSIESPLLTNTTTFDDNTAPTYANRANINSVPSQRQPVVSTPQLKHLQVGKRRQRNPSAIYDSVKEPYSYTTGFHSLTAFIQRRFAPQNTLRIAKALASIRPSFIATTKTLNRDDLIFMEKCFQRTLWEYEDFINACGTPTIVCRRTGEIAAVGKEFSILTGWKKEVLLGKEPNLNINTGGSSGAMSGVTSRGSFTPRTGMDVNPTGRTQPVFLAELLDDESVVEFYEDFAKLAFGDSRGSVMTTCKLLKYKTKADMDMLSGTTSSAGGENEHGAAGNGDVKPENGMGASNGQSQHSLQRQRKWSRGGIAGEAGMNQLGFKDGKVECSYCWTVKRDVFDIPMLIVMNFLPCI

>An_AcuK

METETGKGATAPPAESSGVEQDTAAVGAPADQPPKTNANATSNANGEDQPANGQKANPKDPSRPRRKKARRACFACQRAHLTCGDERPCQRCIKRGLQDACHDGVRKKAKYLHDAPDGALMPGIGGNNFYNNNSMSNGVPSGGINMNGANTVNSAASTQNSSANFYPTPQSNYSLYQENPINHQNSFPSQSPVSPTFSLKTNPTPRNTAPNNNNNNALTSSMPQPATTGVSNAPNQSQNPFAGPFFDPSDPALFNFDLSSMNFENRYGALEFGMLGHMATGAGDSPDSGTHRGSMGRSGSTQFASTPIGGTTTFGESPQNQQPFMFGDPLLNEWPSGQTSGQPHVNVGVYPQSSQGNVIPGHLSKPDAPHAFAIESGPNNFTSPGAATSPQINSGGYEDANAFNNVVTKSNGLSVNGQQRPPTISTPSLKHQSLQMNKRRHRNPSAVYESVKEPYAYTSRFHSLTAFIQRRFSPQKTLQIAKALASIRPSFIATTKTLNRDDLIFMEKCFQRTLWEYEDFINACGTPTIVCRRTGEVAAVGKEFSILTGWKKEVLLGKEPNYNVNTGGSSAANSRNITPRSSVESTGRPHPVFLAELLDDDSVVEFYEDFARLAFGDSRGSVTTRCKLLKYKTKEDMEAAQSDDNGQRWNNHLRKGGIANEAGMNQLGFKDGKVECAYCWTVKRDVFDIPMLIVMNVRLPLP

>Afl_KAB8239936.1

MPGIGGNFYNNTMRSNLPLSRNGANAVNATTQPSSSPNFYPTPQSNSYSVYQENTMNQNSFTSQSPVSPTFTLKANPAARNNSLSSQVNQQPPSTGVSGATNPSQNPFAGPFFDPSDPALFNFDLSSMNFENRYGALEFGMLGHMATGAGDSPSDSATQRGSMGRSGSAQFSGTPITGAAAFGESPGGQQPFIFGDPLLNEWSSGQPTGQTHVNVGGVYPQSGQGSVIPGHLTKADAPHAFAIESGPGSFASPNATTSPQITTGFDDATFSSAVTAKSNGLSANGPRPTITTPSLKHQNLQVGVRRRQRNPSSIYENVKEPYAYTNRFHNLTAFIQRRFSPQKTLQIAKALASIRPSFIATTKTLNRDDLIFMEKCFQRTLFEYEDFINACGTPTIVCRRTGEIAAVGKEFSILTGWKKDVLLGKEPNLNVNTGGSVPGSGTSSRSFTPRGSVAESTPGRPQPVFLAELLDDDSVVEFYEDFARLAFGDSRGSVMTTCKLLKYKTKEDMENQSDDNQRWNSHLRKGGIASEAGMNQLGFKDGKVECAYCWTVKRDVFDIPMLIVMNFLPCI

>Af_AcuK

MKTEVNGSAPALAGDHGGVDQDTPDAGDRTEQAKHKTNGATENAPKSANAKDPSRPRRKKARRACFACQRAHLTCGDERPCQRCIKRGLQDACHDGVRKKAKYLHDAPDGALMPGVGGNFYNNAMRNNMPLSRNGTTTVNTTTQQNSGSNYYPTPQSNSYNVYQDTPLTQNSFPSQSPVSPTFNMKTTPTARSNSLSSSVNQQPPSTTVSGATQSQNPFAGPFFDPSDPALFNFDLSSMNFENRYGALEFGMLGHMATGAGDSPTDSATQRGSIGRSGSTQYSTTPLTGAPGFGESPGNQQPFLFGNDPLLNEWPNSQAPNQGHLNVSGVYPQGGMMHMAKSDAPHAFAIESGPASFSSPSATTSPHINSGHDESSLSNAAVNKSTGLTANGQRPAITTPSLKHQSLQFGVKRRQRNPSTVYESVKEPYAYTNRFHNLTAFIQRRFSPQKTLQIAKALASIRPSFIATTKTLNRDDLIFMEKCFQRTLWEYEDFINACGTPTIVCRRTGEIAAVGKEFSILTGWKKDVLLGKEPNLNVNTGGSSAPGSGNTSRGSFTPRSSTLETATPGRPQPVFLAELLDDDSVVEFYEDFARLAFGDSRGSVMTTCKLLKYKTKEDMELAQSDDNQRWNNHLRKGGIAGEAGMNQLGFKDGKVECAYCWTVKRDVFDIPMLIVMNVSGQCIWTSRHMLISTTVLTVHMTESHTCWWFAPGCPWNALDVLLIILCLFFFFFFFFFFFF

>Ani_EHA23613.1

MNAEPKEQDSPAPSAERTEASQEISAAGAQADKPKTEANGDGTANGASANGQKPNPKDPSRPRRKKARRACFACQRAHLTCGDERPCQRCIKRGLQDACHDGVRKKAKYLHDAPDGALMPGIGGTFYNNPMRNSLPLSRNGANAVNATGQQSAGANFYPTPQSTTYVYQENTINQGSFPSQSPVSPTFNLKATPTARTNSLSSVNPQPPSTSVSGPPGQGQNPFAGPFFDPSDPALFNFDLSSMNFENRYGALEFGMLGHMATGAGDSPSDSATQRGSMGRSGSAQYASTPITGAPGFGESPGNQQPFMFGDPLLNEWPSGQAPGQPHLPGVYPQSGQGSAIPGHLSKADAPHAFAIESGPASFNSPGATTSPQMTTGLEETPFHSAVASKSNGLAPHGQRPMITTPSLKHQNLQVGVRRRQRNPSAIYDSVKEPYAYTSRFHGLTAFIQRRFPPQKTLQIAKALASIRPSFIATTKTLNRDDLIFMEKCFQRTLWEYEDFINACGTPTIVCRRTGEIAAVGKEFSILTGWKKDVLLGKEPNLNVNTGGSSMPNSGASSRSFTPRSTVDNTPGRPQPVFLAELLDDDSVVQFYEDFARLAFGDSRGSVMTTCKLLKYKTKEDMEGAAAEDSQRWNNHLRKGGIASEAGMNQLGFKDGKVECAYCWTVKRDVFDIPMLIVMNVSNTSVPVL

**AcuM homologs**

>An_AcuM

MTEKNTTQPGSAPDQYAGNGTAENTGPSKTLDGNKVGRVDSNAKENAQKSPASKTESTPAGTRTSPKKRRKVNHACVYCRRSHMTCDSERPCTRCIKRNIGHLCHDEPREPSKRSRSEHEQSAADEEGSSNNEYSKVHAMPRKVDIQDAAGQQILADGSLGLTPSSMNAVQPGPMSSSTSQNMSVTSQQQLLGYNDWVGGQNQFQDMHTFHPSYMFNAPEVTNEYNLLGDFLSNSLLDDGSIFQNEDMQRMYSDPTLINSMAVLGGPSTSLLQQSQTLQPQQSQQNQGDTASGATIGNDKARETYYMTAADPAGSDPPEERMNKLLKAKYDAGLLRPFNYVKGYARLSAYMEKNLQVSSRQKIARQLDKFRPKFRDRMQKLTDIELILVEMWFERSLMEYDRVFASMAIPACCWRRTGEIFRGNKEMAELIGVPIELLRDGKLAIHEIIVEDQLVSYWEKFGAIAFDNTQKAMLTSCTLKNPNATSPTEGIPCCFSFTIRRDNHNIPSLIVGNFLPTQRDTK

>Ani_EHA22861.1

MTEKTAASTGPGPDQQTSNGTADRTKPDVTEEEQTGADASAKDNSQNTPSLKADGAAAGAHSSPKKRRKVNHACVYCRRSERPCTRCIKRNIGHLCHDEPREPSKRSRSENDHSAADDEGSSNNEYSGVQMPRNVDGPDAAGQQIIPDGTIGLPTSSVTSVQQPGNMASSGQGLNAQQQMIGYNEWIGGQSQFQDMHTFHPSYMFNAPEVTNEYNLLGDFLSNSLLDDGGVFQNDELQGIYSDPSLLNSMATLGNSNQSLLQQSQPAQPQPNQQAQGEPLQGPTPAVSNDKARETYYMTAADPSGSDPPEERMNKLLKAKYDAGLLRPFNYVKGYARLNQYMERHLQQASRQKILRQLDKFRPKFRERMQSLTDIELILVEMWFERSLMEYDRVFASMAIPACCWRRTGEIFRGNKEMAELIGVPIETLRDGKLAIHEIIVEDQLVSYWEKFGAIAFDNTQKAMLTSCTLKNPNSNSPGDGIPCCFSFTIRRDTHNM

>Af_AcuM

MGCRKSSCPRHHTPKAGTFPPRWFPSAEEPGKKGGQPARPTVVISAGTSEHETITIARRRRTPQLKARHLLSAPANASDLSSSKLIFDVCRSPSDLLLRVPTTNSEHQQAELCATALRPTRSKRLMTENGTAQTGTVPVEQPRNGTMENAKLNMAEGDSSRMESGSKNTASPPVKADNNAAGTHSSPKKRRKVNHACVYCRRSSLYDLLAYDMRFGTTFLIHFLQLASVGCIAGFYLSHLCHDEPREPSKRARSEHEHSTAEEDGHSNNEFSNAQSMPRNVDVQDAAGQQILPDGTVALPPSSVSAVQHNTIPSSSAQNSLGHNSQQLLGYNEWLGGQSQFQDMHTFHPSYMFNAPEVTNEYNLLGDFLSSSLLDDGGMFSNDNLQGIYSDPTLINSMANLDNTALLQQAQPSQPTQSQPHQNDSVQGPSSTVVNDKARETYYMTAADPSGSDPPEERMNKLLKAKYDAGLLKPFNYVKGYARLNQYMEKNMKQSSRQKILRQLDKFRPKFRERMQSLTDIELILVEMWFERSLMEYDRVFASMAIPACCWRRTGEIFRGNKEMAELIGVPIESLRDGKLAIHEIIVEDQLVSYWEKFGAIAFDNTQKAMLTSCTLKNPNSSNPGNGIPCCFSFTIRRDNHNM

>Afl_XP_002379526.1

MTEKNAAQTGTRPEQSGNGTLGNLKNSMAEGDAPKKDSTAKDNSRNSPASKGENAAAGTHASPKKRRKVNHGKQTTSPCIRTRIMAADPHTPLPFRGYELYDANPVATLLACVYCRRSRNIGHLCHDEPREPSKRARSEHEHSVGDDESVQNNDFSNVLQGMTGNVDVQDAAGQQILPNGIPSSSVQHGNLPSSSGQAPGATSQPGKSWILGYNDWLGGQSQFQDMHTFHPSYMFNAPEVTNEYNLLGDFLSNSLLDDGSMFQNDDLQGIYSDPTLIGSMATLGGGPNAALLQQSQPPPLTQTQPSQGESIQGPISGAVNDKARETYYMTAADPSGSDPPEERMNKLLKAKYDAGLLRPFNYVKGYARLNQYMEKNLQQASRQKILRQLDKFRPKFRERMQSLTDIELILVEMWFERSLMEYDRVFASMAIPACCWRRTGEIFRGNKEMAELIGVPIETLRDGKLAIHEIIVEDQLVSYWEKFGAIAFDNTQKAMLTSCTLKSPDSNAPGDGIPCCFSFTIRRDPHNIPSLIVGNFLPSQRKSK

>Cp_XP_003067533.1

MGIMAETAMTKAGEARQPNNGVSGGVTRSTLTEDDAVKAKESAAAKGSQASSAPAEGATVPASASPKKRRKVNHACVYCRRSHMTCDSERPCTRCIKRNIGHLCHDEPRETSRRAKGEQDIQSVEEDGGKANNDFGNNQKMNRKLSGSSINDQLLGDGSIALQSQGGQDISTAAGQVNQQQLLGYNDWPFGLQNQFQDMHTFHPSYMFNAPEVTNEYNLLNDFLSTSLLDESAMYQGDDTPGLYSDMAFMNTMGTNLPGSGPFVQPQQAQQSSMAPPQYLTTSQSQAAQGNAIQRPNSTVGNDKAKETYYMTAADPSGTDPPEERMNKLLKAKYDAGLLKPFNYVNGYARLNKYMEEHLQPASRQKILRQLDKFRPKFRERMQSLTDIELVLVEMWFERSLMEYDRVFASMAIPACCWRRTGQIFRGNKEMAQLIDVPIESLRDGKLAIHEIIVEDQLVSYWEKFGAIAFDSSQKAMLTSCTLKSPDPKSPKQGIQCCFSFTIRRDPHNIPSIIVGNFLPTKRTDR

>Ci_XP_001245779.1

MGIMAETAMTKAGEARQPNNGVSGGVTRSTLTEDDAVKAKESAAAKGSQASSAPAEGATVPASASPKKRRKVNHACVYCRRSHMTCDSERPCTRCIKRNIGHLCHDEPRETSRRAKGEQDIQSVEEDGGKANNDFGNNQKMNRKLSGSSINDQLLGDGSIALQSQGGQDISTAAGQVNQQQLLGYNDWPFGLQNQFQDMHTFHPSYMFNAPEVTNEYNLLNDFLSTSLLDESAMYQGDDTPGLYSDMAFMNTMGTNLPGSGPFVQPQQAQQSSMAPPQYLTTSQSQAAQGNAIQRPNSTVGNDKAKETYYMTAADPSGTDPPEERMNKLLKAKYDAGLLKPFNYVNGYARLNKYMEEHLQPASRQKILRQLDKFRPKFRERMQSLTDIELVLVEMWFERSLMEYDRVFASMAIPACCWRRTGQIFRGNKEMAQLIDVPIESLRDGKLAIHEIIVEDQLVSYWEKFGAIAFDSSQKAMLTSCTLKSPDPKSPKQGIQCCFSFTIRRDPHNIPSIIVGNFLPTKRTDR

>Tm_AcuM

MPETIATTDTDPKIGTLNGGSGGGEEDVKPNVDNASPSQSVNNSGKGSISSNEQHHHLAKADAAAGQAHASPKKRRKVNHACVYCRRSERPCTRCIKRNIGHLCHDEPREPTKKNNRSDHEDSAVIGNEFTNSHNLADAGNQPMLSDSSLSIRPSTIDPSQAVQGNNQQVFGYNAGGSGGGDWAMNGQSNGYQDMHTFHPSYMFNAPEVTNEYNLLGDFLNNSLLDDNGMYGNEELQGLFSDSSLINMSTNINPNAASFAQQQQQQQQLAQLTPQQAANSQANAIQRPASSAVSTDKARENYYMTAADPSGMDPPEERMNKLLKAKYEAGMLRPFNYVKGYARLNQYMESHMQPSLRHKILRQLDKFRPKFRERMHSLTDIELVLSEMWFERSLMEYDRVFASMAIPACCWRRTGEIFRGNKEMAELINVPIENLRDGKLALHQIITEDQLVSYWEKFGAIAFDGSQKAMLTSCTLKSPDDDDSTKGIPCCFSFTIRRDPNAIPALIVGNFLPSERRRHH

>Nc_Aod2

MTGTEATEKPNGKEAGTKDITKSGSDTKPKDHHPTPADDVQKAPKKRRKVNHACLYCRRSHMTCDLERPCTRCIKRNIGHLCHDEPRDTESRKAKSVLGTSTLHDSESQPDIGRNATDKAMRPPGFDSGMGNGSVQVAGAAAVGRGAPLQLVQPGSVAGIQASALGGSMNQFAGLPDSWLTTQNHYHDMHNFHPNYMVAPEVTNEFNLLNEFLSAGLLEESAFMSDDHGLILGANQSAVSGLPNANNNGNNASSNNKGNSSSTSGMLPPSATQGTSMLPPSSDQTTAIGKPASTNLDNARDAYYLQAADPSGNDTPEERMQRLLRAKYEAGLLKPFNYILGYKRLSDYLDGHVSPTSKQKILKQIDRFRPKFREKIQLLTDMDLLMVEMWFERTLLEYDRVFASMAVPACCWRRTGQIFRGNKEMAELIGVPVESLRGGQIALHEILTEESNVRYWEEFGTIAFDPAHDTLITACSLKNPNDDKGTKVVNCCFSFRIRRDDHKIPSLIVGNFLPHDP

>Pa_Rse2

MTATEAMERTAGNETKDIKSESNTKPKDHHAPSTDDQQQHNQHNHHTTHHQTPKKRRKVSHACLYCRRSHMTCDLARPCTRCVKRNIGHLCHDEPRDQGSNKSKSVVAPSTTHGSASQSDLGRGNMNQTAADALRLASFDGSLSSGTGSAASAAFDAAAALGQSNQLQLVQPTAVSGLQGSTLGSSMNQFPHEVSSEFDVLNNYLRANIFEDPVAAPDGQNQGRPIPGFPSSSSMPPPATAPGASLPTANSEQSAAVSKDKTREYYLQAADPSGNADADDRMLQVLTAKVEAGLLQPFNYIKGYQSLQTYLNEHVSPGSRQKILRQLDRFRPKFREKMQGRDHMFLTINEMQIESSLMEYDRVFASMAVPACCWRRTGEIFRGNKEMAELIDVPVEDLRNGKTKLHEILTEESVVRYWEEFGTIAFDPLHDTLLTACTLKSPNKSSKKVVNCCFSFRIKKDNAQVPGLIIGNFIPHDP

>Ca_Cwt1

MSTMSTQKANSSTPGETDSSSSLPPPEITEPTYKKPEKSTKRRKKKLEIACVYCRRSHMICDESRPCQRCIKRGIAHLCYDEPSNSRQRKKAAALRKTQSEGAPMVTSPITLPLTGSTGNQSPLPLPPLQASQSDIRSAPKTENSLQFSQPALHQQQMQENPQGASQSVHTNNTHIYSSQNQQQQPQHQQQLQQQQPQPQPQSRLIKNSVLSQTLPYNQQPFFYSEHANSEFSSLNDFLSMIDDPELVNGALNDDTDGLLNFGVGNNNNSGGSNSNINNTHNNNNNGFSISGGNSTTNLNAVLAYSPNSNLFPPTFGNEADSTQIQSQQQQPELTQQPPALNPVKVEPQAEKTEQQPVISDSARDKFFLTAADPTTEISPEERLKQVIKAKLEAGLLQPYNYAKGYARLQRYMDNYMNISSRQRILKPLSIFRPAFRAIARTLKDVDLVLVEESFERMLLDYDRVFTAMAIPACLWRRTGEIYRGNKEFASLVGVTTDDLKDGKLAIYELMSEESAVNFWEKYGAIAFDKGQKAVLTSCNLRTRDGIKRKSCCFSFTIRRDRYNIPSCIVGNFIPIDP

>Sc_Rds2

MSANSGVKRASKAFKTCLFCKRSHVVCDKQRPCSRCVKRDIAHLCREDDIAVPNEMPSQHESSPNDNNIQGKYANKAHTGIPSDYQNEPVNKSGSTYGEELSPKLDSSLVNDTTSLLLPQQPVFVSENVGSEFSSLNEFLSMLENPLLTQTSLSSSSASNVHLENGSQTTQSPLEYQNDNRRDEIGVARQENRSPTIMSGSSNSISKGDKQDQEKEESRILANANENSAPTPKEQFFLTAADPSTEMTPEHRLKLVINAKLEAGLLKPYNYAKGYARLQDYMDKYMNQSSKQRILKPLSTIRPAFRTIARSLKDVDLVLVEESFERMLLSYDRVFTSMSMPACLCRRTGEIYRANKEFASLVDCTVDDLRDGKLAIYELMTEESAVNFWEKYGSIAFDKGQKAVLTSCSLRTKDGIRKRPCCFSFTIRRDRYNIPICIVGNFIPLS

>Cn_Rds2

MQNNAPSGFQGFHFPLQQSDLYTFAPASQNQQQQQQQQLYYAAQAAHGQQPSTDSSESAGPSQKKKPAGARKHGVKEEPEEHGKADEPKKKRSKRSAGKACVYCRRSHMVCEGGRPCERCIKREIPHLCRDCTPPPHTQQSPHKQEPQQQQQAQQQQSLNQPQVVPSSSQQMPVYADSNFVPSWPLLPDSGAAQIPFGEAPSEQIQNPGDAGIMGPPSLGSSKEDGELAALSKFMKDLGVPNLPNDFLSFMNQLDKPEAGNSLSIASSSDANLFPSSGASQVVGLNKGKGKLGQISRIDKYLMAAADQPNGTRASRLAQVIKAKYDAGLLKPYDYAKGYERMNKWMESGRAAPRMDSRAGSEIPEESPQRSTAAMRNGRLSVAPLGPNVPPFGRSISPESRRRILAALAGFRPKFRQIARTLTNVDLVFVEEAMERWMLEYDRAFASIHTPSCIWRRTGEIQKANQEFSNLTGIPAYMFRDGQLCVYELMDEDSAVRYWEGYAKIAFDPSQRAMSILCTLHIPLSLTRHRPRHLTNANPNNVSKSATSGPPPQAPYTPDLALPQQNMFNDGASSDAGTVIGEEYREMKCAFSVTIRRDAWGVPVAIMGQWIPIQ
